# Physicochemical Characterization and Metabolites Identification of the Synthetic Cannabinoid MDMB-5′Br-PINACA Using *In Silico* and *In Vitro* Approaches

**DOI:** 10.1021/acs.chemrestox.6c00107

**Published:** 2026-05-24

**Authors:** Alexandre B. Godoi, Natalícia J. Antunes, Júlio C. C. da Silva, Gabriel Cordeiro, Tássia F. D. Castro, Jose L. Costa

**Affiliations:** † 28132 Campinas Poison Control Center, Universidade Estadual de Campinas (UNICAMP), Campinas, SP 13083-859, Brazil; ‡ School of Medical Sciences, Universidade Estadual de Campinas (UNICAMP), Campinas, SP 13083-859, Brazil; § Faculty of Pharmaceutical Sciences, Universidade Estadual de Campinas (UNICAMP), Campinas, SP 13083-871, Brazil; ∥ Nova Analítica Imp. Exp. LTDA, Sao Paulo, SP 09941-202, Brazil

## Abstract

MDMB-5′Br-PINACA is a recently identified brominated synthetic cannabinoid that was detected in herbal materials seized in Brazil in 2025, raising concerns regarding further potential intoxication cases. In this sense, the evaluation of physicochemical properties and metabolic fate may improve its analytical detectability. Therefore, an integrated *in silico* and *in vitro* approach was employed to investigate the physicochemical properties and phase I metabolism of MDMB-5′Br-PINACA. Physicochemical parameters and predicted metabolic pathways were first evaluated using BioTransformer 3.0 and XenoSite, providing complementary insights into likely sites of metabolism. *In vitro* metabolism was subsequently assessed using pooled human liver microsomes associated with liquid chromatography coupled to high-resolution mass spectrometry (LC-HRMS) analysis. MS^2^-based molecular networking (MN) was applied as an exploratory and confirmatory strategy to guide metabolite annotation by clustering structurally related features and prioritizing candidates linked to MDMB-5′Br-PINACA. A total of twenty-seven metabolites were level 2 annotated, encompassing aliphatic and aromatic hydroxylation, sequential alcohol oxidation to ketone, aldehyde, and carboxylic acid derivatives, ester hydrolysis, intramolecular lactone formation, and *N*-dealkylation with loss of the pentyl side chain. Hydroxylations of the pentyl chain and *tert*-butyl moiety and secondary oxidative reactions emerged as the predominant pathways under the experimental conditions, in agreement with *in silico* predictions. However, lactone formation was exclusively revealed by *in vitro* experiments, demonstrating limitations of current *in silico* prediction approaches. The integration of computational prediction, LC-HRMS, and MN substantially enhanced metabolite coverage and confidence of structural assignment. These findings provide a detailed metabolic map of MDMB-5′Br-PINACA and underscore the value of combining *in silico* and *in vitro* approaches to improve metabolite identification, supporting forensic and clinical investigations of intoxication involving this synthetic cannabinoid.

## Graphical abstract

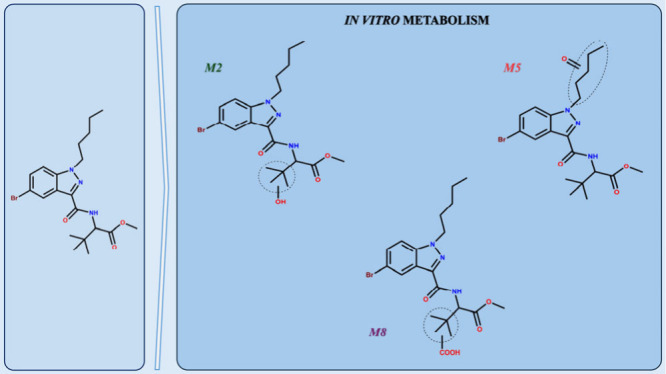

## 1 Introduction

Over the past decade, the emergence of New Psychoactive Substances (NPS) has challenged forensic science, clinical toxicology, and public health worldwide.^1^ These compounds are typically synthesized not only to mimic the psychoactive effects of controlled drugs but also to circumvent existing legislation through structural modifications.^2^ Among the NPS classes, synthetic cannabinoids (SCs) remain the most structurally diverse and dynamic group, accounting for a significant proportion of the novel drugs reported annually by international monitoring programs.^3,4^ These molecules are commonly found in herbal smoking mixtures but have also been detected in e-liquids, tablets, and impregnated paper sheets.^5^ Their high affinity for the cannabinoid type 1 receptor (CB_1_R) often results in more pronounced psychoactive effects, exceeding those of Δ^9^-tetrahydrocannabinol (Δ^9^-THC) and contributing to severe and unpredictable toxicological outcomes.^6–8^

The continuous introduction of novel SC analogs is largely driven by legislative control, pushing clandestine laboratories to design chemically modified structures.^9^ As a result, these newly identified structures lack pharmacological and toxicological data. Halogenated derivatives, particularly brominated SCs, represent a smaller but noteworthy and more recent subgroup within this class. Halogen substitution may increase lipophilicity, blood–brain barrier permeability, receptor binding affinity, and metabolic stability, potentially altering their biological activity.^10,11^ MDMB-5′Br-PINACA, a methyl ester analog structurally related to ADB-5′Br-PINACA, was identified for the first time in 2025, found in seized herbal materials in Brazil.^12^ To date, no experimental data are available regarding its physicochemical, metabolism, or toxicological properties.

Metabolism studies are essential for understanding NPS toxicity, as many of these compounds undergo extensive and rapid metabolism, yielding a wide range of metabolites. The detection of these metabolites is often essential for intoxication diagnosis, as many times the parent compound may be absent from biological specimens depending on the postexposure interval.^13^ Furthermore, certain metabolites can contribute to, or even be primarily responsible for, the observed toxic effects.^7^ Therefore, liver microsome assays are widely applied to predict metabolic stability, clearance, enzyme kinetics, and metabolic pathways, enabling interspecies comparison and supporting toxicological interpretation.^14^ Pooled human liver microsomes (pHLM) are commonly used to evaluate both qualitative and quantitative aspects of xenobiotic metabolism, providing a relevant *in vitro* model for the identification of human-specific metabolic pathways.^15^ Complementary *in silico* tools have significantly enhanced metabolite identification, allowing rapid and simple prediction of potential phase I and phase II metabolites prior to experimental work.^7,16^

For a more comprehensive understanding of metabolites related to NPS metabolism, liquid chromatography coupled with high-resolution mass spectrometry (LC-HRMS) stands out as the most comprehensive analytical technique for this type of application. However, nontargeted LC-HRMS approaches generate a large volume of data and, consequently, forensic laboratories require appropriate software and specialized personnel for the efficient interpretation of this information.^17,18^ In this context, molecular networking (MN) emerges as an innovative strategy that combines high-resolution mass spectrometry with advanced data processing to investigate metabolic pathways.^19–22^

Here, we provided an integrated characterization of MDMB-5′Br-PINACA by combining *in silico* prediction with *in vitro* phase I metabolism in pHLM followed by LC-HRMS analysis. We further applied MS^2^-based MN to systematically prioritize and annotate related metabolite features. Therefore, this work aimed to characterize physicochemical properties of this new SC, and to experimentally establish its phase I metabolic profile to support toxicological screening and interpretation.

## 2 Materials and Methods

### 2.1 Chemical and Reagents

LC-MS grade methanol and LC-MS grade water, acetonitrile, glucose-6-phosphate, magnesium chloride hexahydrate, sodium citrate tribasic dihydrate, β-nicotinamide adenine dinucleotide phosphate hydrate (NADP^+^), and pooled human liver microsomes (pHLM) at 20 mg/mL were purchased from Sigma-Aldrich (St. Louis, MI, USA). Ultrapure water was obtained using a Mili-Q RG system, Milipore (Burlington, MA, USA). Formic acid was acquired from Scharlab (Barcelona, Spain) and Gentest 0.5 M phosphate buffer pH 7.4, glucose-6-phosphate dehydrogenase from Corning (Woburn, MA, USA). Due to the absence of commercially available certified reference material, MDMB-5′Br-PINACA was obtained by purification of an herbal seized sample according to a method previously developed and described by our group,^12^ resulting in a limited amount of compound. Subsequently, MDMB-5′Br-PINACA was prepared as a stock solution at 2 mg/mL (4.58 mM). N-ethyl pentedrone (NEP) hydrochloride reference material, a positive control for the incubations, was acquired from Cayman Chemical (Ann Arbor, MI, USA) and a stock solution prepared at 1 mg/mL in methanol.

### 2.2 In Silico Prediction of MDMB-5′Br-PINACA Physicochemical Properties and Metabolism

ChemDraw Ultra (Revvity Signals Software, version 14.0) was employed for drawing the molecular structure of MDMB-5′Br-PINACA and then converting to SMILES format for further *in silico* analysis. For *in silico* prediction of MDMB-5′Br-PINACA physicochemical properties, SwissADME^23^ was employed, also providing pharmacokinetic features associated with its absorption, distribution, metabolism, and excretion (ADME) properties, as well as its blood–brain barrier (BBB) penetration and affinity for P-glycoprotein (P-gp). Furthermore, BIOLED-Egg^24^ tool was also used to determine the lipophilicity and polarity of the studied compound.

For MDMB-5′Br-PINACA *in silico* metabolites prediction, BioTransformer 3.0 ^25^ and XenoSite^26^ were employed. For BioTransformer 3.0 server, both Phase I (cytochrome P450 - CYP450) Transformation and Phase II Transformation were set, using a combination between rule-based method and machine-learning models. Two reaction iterations were defined. For XenoSite analysis, phase I reactions, as well as epoxidation, quinonation, *N*-dealkylation, and uridine diphosphate-glucuronosyltransferase (UGT) conjugation were selected for predicting the main sites of metabolism (SOM).

### 2.3 Microsomal Incubation

All incubations followed the good practices guideline for metabolism studies^27^ and were based on previously published studies from our group.^14,28^ For phase I metabolite elucidation, 20 microliters of 4.58 mM MDMB-5′Br-PINACA were diluted into 780 μL of a NADPH-regenerating system (1.1 mM NADP^+^, 10 mM glucose-6-phosphate, 1 U/mL glucose-6-phosphate dehydrogenase, 5 mM sodium citrate and 66 mM magnesium chloride in 100 mM phosphate buffer, pH 7.4) in a 1.5 mL propylene tube. Aliquots of 100 μL were transferred to new tubes and preincubated for 5 min in an MTC 100 thermo shaker incubator (Miulab, Hangzhou, ZJ, China) at 300 rpm and 37 °C. Reactions were started by adding 100 μL of pHLM at 5 mg/mL into the preincubated shaking tubes (MDMB-5′Br-PINACA and microsomal protein final concentrations of 57.25 μM and 2.5 mg/mL, respectively). After 0, 30, and 60 min, metabolism reactions were interrupted by adding 400 μL ice-cold acetonitrile. The samples were mixed in a BenchMixer XL (Benchmark, NJ, USA) for 5 min and centrifuged at 12,000 × *g* for 15 min at 4 °C (Hettich Universal 320 R, Tuttlingen, BW, Germany). Finally, supernatants were transferred to vials (200 μL) and 10 μL was injected into a LC-HRMS system. Positive controls were prepared by incubating NEP under the same condition as MDMB-5′Br-PINACA (drug concentration of 57.25 μM and microsomal protein concentration of 2.5 mg/mL) to achieve the suitability of pHLM based on previous studies.^14^ Negative controls were also prepared by incubating the synthetic cannabinoid in buffer solution, in the absence of microsome, and cofactor solutions. All incubations were performed in a single replicate, as the objective of this study was qualitative metabolite profiling rather than quantitative assessment. Given the exploratory nature of this work, results are intended for qualitative/semiquantitative metabolite profiling rather than quantitative comparison. Furthermore, the limited availability of the purified compound also constrained the number of replicates performed.

### 2.4 Detection of MDMB-5′Br-PINACA and Metabolites by LC-HRMS

The identification of MDMB-5′Br-PINACA and metabolites was performed using a Vanquish Horizon Binary UHPLC chromatographic system coupled to an Orbitrap Exploris 120 mass spectrometry (Thermo Scientific, Bremen, Germany) using electrospray ionization (ESI) source operating in positive mode. Chromatographic separation was performed using a Luna Omega column (Phenomenex, 2.1 × 100 mm, 1.6 μm) at 40 °C. Mobile phases were 0.1% formic acid in LC-MS grade water (MPA) and 0.1% formic acid in LC-MS grade methanol (MPB) at a flow rate of 0.3 mL/min. The chromatographic scheme was performed in gradient, starting with 10% MPB for 1 min, ramping to 95% MPB for up to 17 min, maintaining this proportion for 5 min and finally returning to the initial condition for up to 22.1 min, maintaining this proportion for column re-equilibrium until 26 min. Data were acquired in full MS^1^ scan mode at a resolution of 120,000, followed by MS/MS experiments (MS^2^) top-4 acquisition at a resolution of 15,000, using stepped normalized collision energy (NCE = 18.75, 37.50, and 56.25) in combination with a dynamic exclusion list to maximize spectral coverage during MS/MS acquisition. Internal mass calibration was achieved using the EASY-IC system, ensuring high mass accuracy throughout the entire analysis.

### 2.5 LC-HRMS Data Analysis and Interpretation

To describe and characterize MDMB-5′Br-PINACA and its metabolites incubated in pHLM, all acquired chromatograms and mass spectra were analyzed using Compound Discoverer software version 3.4 (Thermo Scientific, Bremen, Germany). Initially, the raw (.RAW) files were imported into the software, and chromatographic alignment across samples was performed as the initial step of the workflow. This step corrected retention time variations between analytical runs and ensured consistent feature matching across the entire data set. After alignment, feature detection was carried out from MS^1^ data through extracted ion chromatograms (XICs), using a maximum mass tolerance of 5 ppm for MS^1^. Next, the detected features underwent empirical minimal formula prediction based on accurate mass, isotopic pattern, and elemental constraints defined within the workflow. Subsequently, the software grouped related signals and removed redundant data, consolidating adducts, isotopologues, and in-source fragments into a single putative compound. The presence of these features was evaluated across all aligned samples, which ensured proper assessment of compounds detected in individual samples. This process generated a final matrix of aligned and comparable features suitable for interpretative analyses. Finally, MS^2^ data associated with each feature supported spectral similarity analyses and enabled construction of MS^2^-based MN. In this approach, fragmentation spectra with similar patterns formed network connections that reflected structural relationships among compounds. Visualization of the molecular network facilitated identification of related compounds and potential metabolites. Metabolite identification was performed according to widely accepted metabolomics guidelines, corresponding to level 2 annotation - putatively annotated compounds.^29^ Structural proposals were based on high-resolution accurate mass measurements, isotopic pattern, and MS^2^ fragmentation data. Since authentic reference standards for the detected metabolites are not yet commercially available, it was not possible to match retention times. Therefore, definitive structural confirmation (level 1) was not achieved. To ensure confidence in the proposed annotations, strict criteria were applied, including mass errors below 5 ppm for precursor ions and below 10 ppm for fragment ions, as well as the presence of at least two consistent and structurally informative product ions in the MS^2^ spectra. Despite these measures, the exact positions of metabolic modifications, particularly in the case of positional isomers, should be considered as tentative. The percentages of metabolite formation in pHLM (metabolites relative area) were calculated using each metabolite absolute area of the chromatographic peaks normalized by the summed area of all detected metabolites.

## 3 Results and Discussion

### 3.1 In Silico Prediction of MDMB-5′Br-PINACA Physicochemical Properties

Exploratory physicochemical and pharmacokinetic properties of MDMB-5′Br-PINACA, predicted based on its molecular structure were summarized in Figure 1. Based on SwissADME *in silico* analysis, MDMB-5′Br-PINACA displayed physicochemical properties consistent with high drug-like potential. The compound showed a topological polar surface area (TPSA) of 73.22 Å^2^, with four hydrogen bond acceptors and one hydrogen bond donor, parameters generally associated with passive membrane permeability. Lipophilicity values showed consensus log*P* of 4.17 (ranging from 3.18 to 5.28 across different models). Water solubility predictions indicated low solubility (−5.47 for ESOL, – 6.57 for Ali, and – 6.17 for SILICOS-IT models). The moderate TPSA and balanced hydrogen-bonding capacity favor membrane permeability, while the high consensus log*P* value is consistent with strong lipophilicity, facilitating BBB penetration.^30^ Notably, the compound is not expected to be a substrate of P-gp, which suggests reduced susceptibility to active efflux from the central nervous system (CNS), thereby enhancing brain exposure. This characteristic may contribute to a more pronounced psychoactive profile, potentially enhancing brain exposure and warranting further investigation of neurotoxicity.

**Figure 1:**
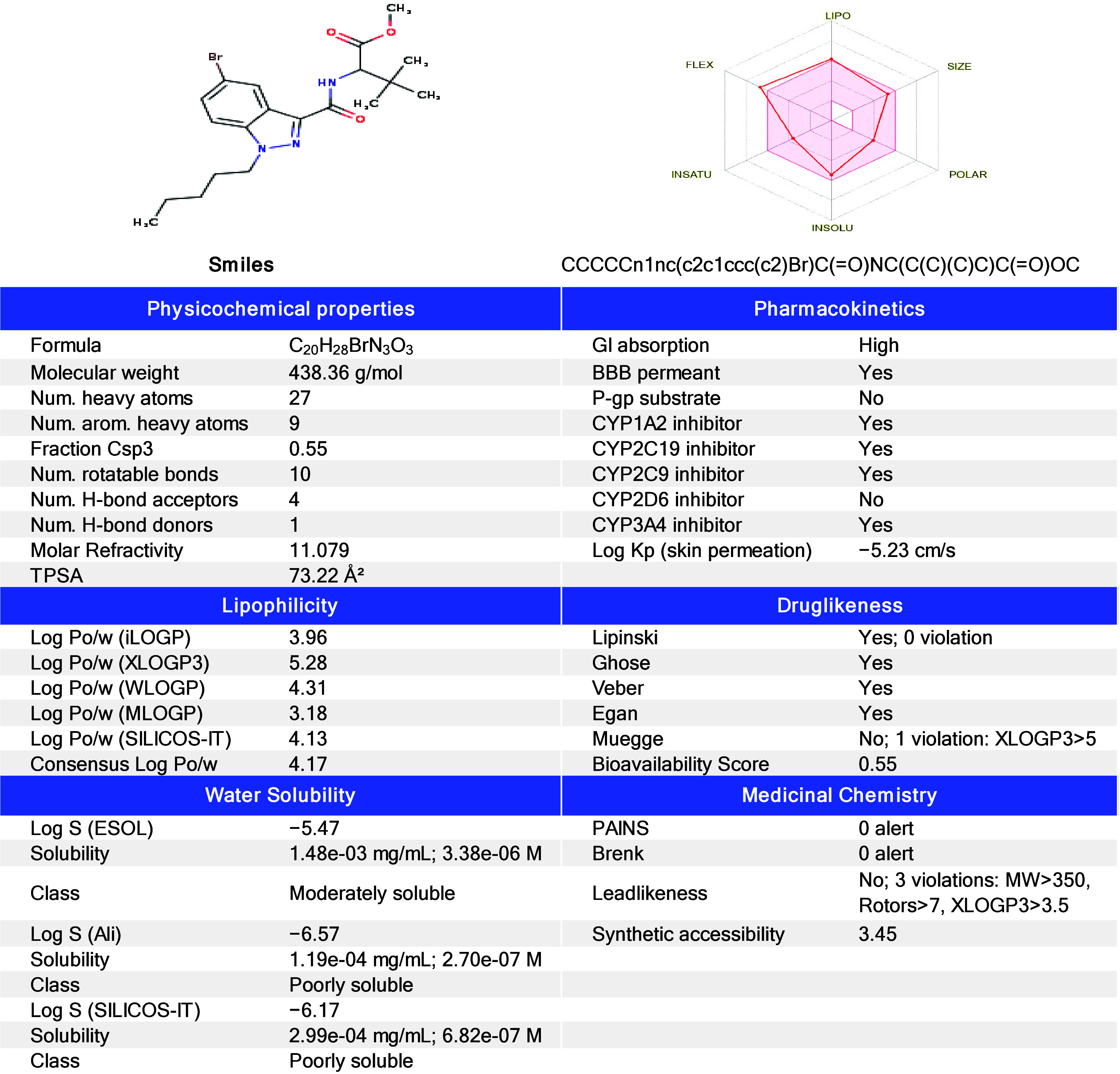
*In silico* prediction of the physicochemical and pharmacokinetic properties of MDMB-5′Br-PINACA using SwissADME. TPSA, topological polar surface area; GI, gastrointestinal tract; BBB, blood-brain barrier; P-gp, P-glycoprotein; CYP, cytochrome P450.

Furthermore, assessing ADME parameters is essential to early drug research and toxicological investigations.^31^ Pharmacokinetic predicted parameters suggested high gastrointestinal absorption and the ability to cross the BBB, with no evidence of being a P-gp substrate. The compound was predicted to inhibit CYP1A2, CYP2C19, CYP2C9, and CYP3A4, but not CYP2D6. This data highlights the potential for drug–drug interactions, which is clinically relevant considering the frequent coadministration of several psychoactive substances.^32^ Previous work demonstrated important drug–drug interactions associated with other SC, such as JWH-073 and JWH-018.^33^ Additionally, the bioavailability provided by the Abbott Bioavailability Score was estimated at 0.55.^34^ The low aqueous solubility predicted by multiple models may limit oral bioavailability and contribute to variable absorption, although the bioavailability score indicates a moderate probability of systemic distribution upon ingestion.

Regarding druglikeness, the compound satisfied the Lipinski, Ghose, Veber, and Egan rules, but showed one violation of the Muegge filter (XLOGP 3 > 5). Leadlikeness filters indicated three violations (MW > 350, rotatable bonds >7, and XLOGP 3 > 3.5). No PAINS or Brenk alerts were detected, and the synthetic accessibility score was considered from low to moderate (3.45). The absence of multiple violations in druglikeness and medicinal chemistry filters suggests that MDMB-5′Br-PINACA retains structural characteristics associated with favorable pharmacokinetics, such as adequate absorption and metabolic stability.^35,36^ In the context of NPS, these features may also imply a prolonged duration of action and an enhanced ability to reach effective concentrations in the CNS.

Additionally, *in silico* analysis predicted moderate synthetic accessibility of MDMB-5′Br-PINACA, as it aligns with recent reports emphasizing the ease with which new SCRA analogues can be generated through minor structural modifications.^10,37^ Indeed, MDMB-5′Br-PINACA has been highlighted as a potential emerging cannabinoid due to its straightforward synthesis and the combinatorial possibilities of precursor substitution.^10^ This synthetic feasibility lowers the threshold for clandestine production and accelerates the introduction of novel compounds into the drug market, reinforcing the need for proactive monitoring and toxicological characterization to anticipate its potential emergence.

Altogether, the predicted physicochemical and pharmacokinetic profile of MDMB-5′Br-PINACA supports its potential for central activity, in agreement with previously reported synthetic cannabinoids.^38,39^ Overall, these *in silico* predictions should be considered an initial and exploratory assessment aimed at providing complementary information on MDMB-5′Br-PINACA, an emerging SCRA recently described in the literature. Although computational tools such as SwissADME offer valuable insights into physicochemical behavior and pharmacokinetic tendencies, they do not replace experimental evidence, but rather support hypothesis generation and early risk assessment.

### 3.2 In Silico Prediction of MDMB-5′Br-PINACA Metabolites

The *in silico* metabolism prediction performed using BioTransformer 3.0 indicated that MDMB-5′Br-PINACA is highly susceptible to extensive metabolism by CYP450 enzymes. A total of 52 putative metabolites were predicted for the parent compound, reflecting a broad range of phase I and subsequent secondary oxidative transformations.

The most prevalent metabolic reaction predicted by BioTransformer 3.0 was hydroxylation, occurring at multiple regions of the molecule, including in the pentyl side chain, with preferential modification of terminal, penultimate, and antepenultimate secondary carbons, as well as aromatic hydroxylation on the brominated indazole ring system in the *ortho* and *meta* positions to the bromine substituent. Moreover, the addition of the hydroxyl group was also predicted to the *tert*-butyl and terminal methyl-ester moieties.

In addition to hydroxylation, terminal desaturation of the *N*-alkyl chain was also predicted, leading to the formation of a terminal alkene-containing metabolites. These unsaturated intermediates were further implicated in secondary transformations, including epoxidation reactions. Several other secondary metabolites were described, including oxidations of the primarily hydroxylated intermediates, leading to both aldehyde and ketone derivatives, and also sequential hydroxylations. No phase II metabolites were predicted by BioTransformer 3.0 when only the parent structure was used as input.

Complementary *in silico* analysis using XenoSite provided a site-specific and reaction-oriented prediction of MDMB-5′Br-PINACA metabolism, with metabolic liability visualized through color-coded probability maps. In this model, darker and warmer colors indicate a higher confidence of metabolic transformation at a specific molecular site. The phase I metabolism prediction highlighted several SOM. The pentyl side chain displayed strong probabilities for stable and unstable oxidation, particularly at terminal and subterminal carbons. Additionally, oxygenation reactions were also likely to occur in the primary carbons of the *tert*-butyl moiety, supporting the possibility of further subsequent oxidation to aldehyde or ketone intermediates. In contrast, dehydrogenation, quinonation, and reduction reactions were predicted to have low probability across all molecular regions, with no major hotspots identified for these pathways.

Hydrolysis reactions were also predicted by the platform, with a high likelihood of ester bond cleavage. In particular, the methyl ester moiety of the tert-leucinate side chain was identified as a prominent site for hydrolysis, suggesting rapid conversion to the corresponding carboxylic acid. Moreover, hydrolysis of the adjacent amide linkage connecting the indazole core to the amino acid-derived side chain was also predicted, even though with lower probability.

The indazole aromatic system was identified as a site for epoxidation. These predictions suggest the possible formation of arene oxide intermediates, which may rearrange to phenolic metabolites or undergo further detoxification pathways. *N*-dealkylation was predicted at two distinct nitrogen-containing regions of MDMB-5′Br-PINACA. High-probability hotspots were localized at the indazole *N*-alkyl substituent linking the heteroaromatic core to the aliphatic side chain, as well as at the amide nitrogen within the carboxamide linkage of the amino acid–derived moiety.

Regarding phase II metabolism, UGT-mediated conjugation was predicted at the indazole ring, with *N*-glucuronidation showing higher probability at the pyridine-like N1 position and lower probability at the pyrrole-like N2 nitrogen, as indicated by the colored-probability map. Both *in silico* tools evaluated phase II glucuronidation reactions exclusively based on the initially input parent structure. Consequently, neither BioTransformer 3.0 nor XenoSite assessed UGT-mediated conjugation following phase I metabolic transformations, as prior functionalization by oxidative or hydrolytic reactions was not considered. Under these conditions, the predictions are restricted to conjugation reactions directly accessible on the parent compound.

The combined use of BioTransformer 3.0 and XenoSite provided a complementary and internally consistent overview of the predicted metabolic fate of MDMB-5′Br-PINACA (Figure 2). Notably, both platforms converged on oxidative phase I metabolism as the dominant pathway, reinforcing the high metabolic liability of this synthetic cannabinoid. Nevertheless, this may also represent an inherent limitation of these *in silico* metabolism prediction tools when phase II reactions are assessed solely from the parent structure. In many cases, phase I metabolism is essential to introduce functional groups that enable subsequent conjugative metabolism. Phase I predictions indicate the formation of multiple hydroxylated and carboxylic acid–containing intermediates. These functionalized metabolites would be chemically compatible with subsequent UGT-mediated glucuronidation. Incorporation of these functionally modified intermediates into the prediction workflow would likely expand the range of phase II conjugates identified.

**Figure 2:**
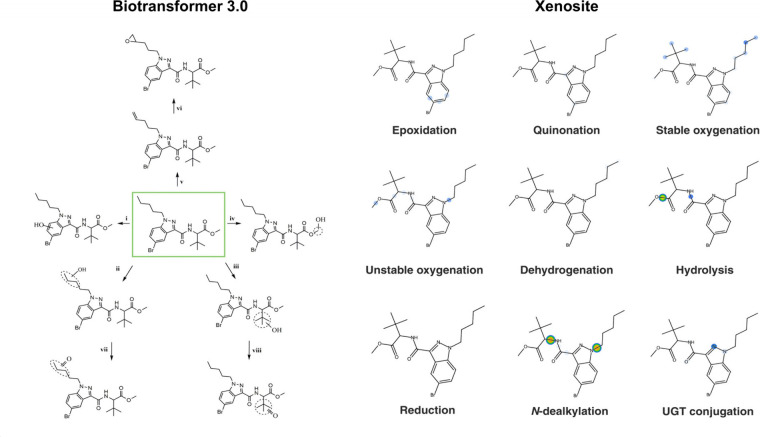
*In silico* prediction of the metabolism of MDMB-5′Br-PINACA using BioTransformer 3.0 and Xenosite. Predicted metabolic reactions by BioTransformer 3.0 were represented as such **i**) aromatic hydroxylation, **ii**) hydroxylation in pentyl side chain, **iii**) hydroxylation in *tert*-butyl moiety, **iv**) hydroxylation in terminal methyl-ester moiety, **v**) terminal desaturation, **vi**) epoxidation, **vii**) alcohol oxidation to ketone or aldehyde in pentyl side chain, **viii**) alcohol oxidation to ketone or aldehyde in *tert*-butyl moiety.

Regarding phase I metabolism, a strong agreement between the two tools was observed for aliphatic oxidation of the pentyl side chain, which emerged as a primary site of metabolism. Both BioTransformer 3.0 and XenoSite consistently predicted hydroxylation at terminal and subterminal carbons. Similarly, oxidative modifications of the *tert*-butyl moiety were predicted by both platforms, indicating susceptibility of this region to oxygenation and subsequent secondary oxidation to carbonyl-containing metabolites. Another point of convergence was the prediction of aromatic metabolism at the indazole ring system, in which both platforms predicted aromatic hydroxylation at *ortho* and *meta* positions to the bromine substituent. Both tools also indicated the formation of secondary oxidative metabolites, including sequential hydroxylations and further oxidation of alcohol intermediates to aldehydes or ketones. The consistency of these predictions across platforms strengthens the hypothesis of multistep oxidative metabolism for MDMB-5′Br-PINACA.

Despite substantial overlap, each platform provided distinct and tool-specific insights. BioTransformer 3.0 predicted a substantial number of metabolites derived from multistep and combinatorial transformations, including extensive sequential oxidations. This reflects BioTransformer’s rule-based approach, which emphasizes metabolic breadth and cumulative reactions, which is important and valuable considering *in vitro* and *in vivo* scenarios. In contrast, XenoSite offered a considerably broader metabolite space with a wider diversity of reactions. This platform also provides a site-specific and probability-weighted perspective, highlighting regions of highest metabolic susceptibility - without explicitly enumerating downstream metabolites.

Notably differences were highlighted in epoxidation reactions so that this transformation was associated with distinct molecular regions and mechanistic contexts. BioTransformer 3.0 predicted epoxidation as a secondary event following terminal desaturation of the pentyl side chain, resulting in the formation of a C4–C5 double bond that serves as a precursor for epoxide formation. In contrast, XenoSite exclusively predicted epoxidation within the indazole aromatic ring. Additionally, terminal desaturation of the pentyl chain was strongly supported by BioTransformer 3.0, whereas XenoSite predicted this reaction with very low probability, as reflected by weak color intensities in the dehydrogenation probability maps. Hydrolysis and *N*-dealkylation reactions were uniquely emphasized by XenoSite, suggesting a potentially important nonoxidative clearance pathway that was not prominently featured in BioTransformer 3.0 predictions. Regarding phase II metabolism, BioTransformer 3.0 did not predict conjugative metabolites when only the parent compound was considered, whereas XenoSite identified direct *N*-glucuronidation of the indazole ring. These data highlight methodological differences between the platforms and also suggest a broader extension of the metabolic landscape of Xenosite.

### 3.3 Identification of MDMB-5′Br-PINACA Phase I Metabolites Using pHLM

Using a molecular network-based metabolite search, MDMB-5′Br-PINACA and twenty-seven phase I metabolites were level 2 annotated following incubation with pHLM in the presence of an NADPH-regenerating system (Table 1). It is important to note that incubations were performed in a single replicate, as the objective of this study was qualitative metabolite profiling rather than quantitative kinetic assessment. Therefore, the relative peak areas reported herein should be interpreted as semiquantitative indicators of metabolite prevalence under the experimental conditions. Precursor assignment was based on MS^1^ accurate mass data (Figure S1). Metabolite identification was based on precursor and fragments exact mass, characteristic isotopic patterns, retention time differences, and detailed MS^2^ fragmentation analysis (Figure S2), all supported by molecular networking visualization.

**Table 1:** Molecular Characterization of MDMB-5′Br-PINACA and Metabolites after Incubation in Pooled Human Liver Microsomes (pHLM)

Molecule	Metabolic reaction	Fragment	Molecular formula	Theorical exact mass [M + H]^+^	Measured exact mass [M + H]^+^	Mass error (ppm)	Retention time (min)
MDMB-5′Br-PINACA	-	-	C_20_H_28_BrN_3_O_3_	438.1387	438.1384	–0.68	17.67
		F1	C_19_H_24_BrN_3_O_2_	406.1125	406.1122	–0.74	
		F2	C_18_H_24_BrN_3_O	378.1176	378.1177	0.26	
		F3	C_13_H_13_BrN_2_O	293.0284	293.0284	0.00	
		F4	C_8_H_3_BrN_2_O	222.9502	222.9502	0.00	
		F5	C_5_H_8_	69.0699	69.0698	–1.45	
M1a	Aliphatic hydroxylation in pentylic chain	-	C_20_H_28_BrN_3_O_4_	454.1336	454.1337	0.22	15.91
		F6	C_19_H_24_BrN_3_O_3_	422.1074	422.1049	–5.92	
		F7	C_18_H_24_BrN_3_O2	394.1125	394.1123	–0.51	
		F8	C_13_H_13_BrN_2_O_2_	309.0233	309.0233	0.00	
		F4	C_8_H_3_BrN_2_O	222.9502	222.9500	–0.90	
		F5	C_5_H_8_	69.0699	69.0699	0.00	
M1b	Aliphatic hydroxylation in pentylic chain	-	C_20_H_28_BrN_3_O_4_	454.1336	454.1337	0.22	15.98
		F6	C_19_H_24_BrN_3_O_3_	422.1074	422.1078	0.95	
		F7	C_18_H_24_BrN_3_O_2_	394.1125	394.1125	0.00	
		F8	C_13_H_13_BrN_2_O_2_	309.0233	309.0234	0.32	
		F4	C_8_H_3_BrN_2_O	222.9502	222.9502	0.00	
		F5	C_5_H_8_	69.0699	69.0700	1.45	
M1c	Aliphatic hydroxylation in pentylic chain	-	C_20_H_28_BrN_3_O_4_	454.1336	454.1337	0.22	16.09
		F6	C_19_H_24_BrN_3_O_3_	422.1074	422.1056	–4.26	
		F7	C_18_H_24_BrN_3_O_2_	394.1125	394.1112	–3.30	
		F8	C_13_H_13_BrN_2_O_2_	309.0233	309.0233	0.00	
		F4	C_8_H_3_BrN_2_O	222.9502	222.9500	–0.90	
M2	Aliphatic hydroxylation in *tert*-butyl moiety	-	C_20_H_28_BrN_3_O_4_	454.1336	454.1336	0.00	16.23
		F9	C_19_H_24_BrN_3_O_3_	422.1074	422.1073	–0.24	
		F10	C_18_H_24_BrN_3_O2	394.1125	394.1119	–1.52	
		F11	C_17_H_22_BrN_3_O	364.1019	364.1020	0.27	
		F3	C_13_H_13_BrN_2_O	293.0284	293.0282	–0.68	
		F4	C_8_H_3_BrN_2_O	222.9502	222.9500	–0.90	
M3	Aromatic hydroxylation	-	C_20_H_28_BrN_3_O_4_	454.1336	454.1336	0.00	17.28
		F12	C_19_H_24_BrN_3_O_3_	422.1074	422.1076	0.47	
		F13	C_18_H_24_BrN_3_O_2_	394.1125	394.1124	–0.25	
		F14	C_13_H_13_BrN_2_O_2_	309.0233	309.0233	0.00	
		F15	C_8_H_3_BrN_2_O_2_	238.9451	238.9450	–0.42	
		F5	C_5_H_8_	69.0699	69.0699	0.00	
M4a	Aliphatic hydroxylation in pentylic chain + Aliphatic hydroxylation in *tert*-butyl moiety	-	C_20_H_28_BrN_3_O_5_	470.1285	470.1287	0.43	13.85
		F16	C_19_H_24_BrN_3_O_4_	438.1023	438.1018	–1.14	
		F17	C_19_H_22_BrN_3_O_3_	420.0917	420.0910	–1.67	
		F18	C_17_H_22_BrN_3_O_2_	380.0968	380.0965	–0.79	
		F8	C_13_H_13_BrN_2_O_2_	309.0233	309.0230	–0.97	
		F4	C_8_H_3_BrN_2_O	222.9502	222.9497	–2.24	
		F5	C_5_H_8_	69.0699	69.0699	0.00	
M4b	Aliphatic hydroxylation in pentylic chain + Aliphatic hydroxylation in *tert*-butyl moiety	-	C_20_H_28_BrN_3_O_5_	470.1285	470.1285	0.00	14.27
		F16	C_19_H_24_BrN_3_O_4_	438.1023	438.1021	–0.46	
		F17	C_19_H_22_BrN_3_O_3_	420.0917	420.0937	4.76	
		F18	C_17_H_22_BrN_3_O_2_	380.0968	380.0995	7.10	
		F8	C_13_H_13_BrN_2_O_2_	309.0233	309.0226	–2.27	
		F4	C_8_H_3_BrN_2_O	222.9502	222.9502	0.00	
		F5	C_5_H_8_	69.0699	69.0700	1.45	
M5a	Aliphatic hydroxylation in pentylic chain + Alcohol oxidation into ketone or aldehyde	-	C_20_H_26_BrN_3_O_4_	452.1180	452.1179	–0.22	15.51
		F19	C_18_H_22_BrN_3_O_2_	392.0968	392.0968	0.00	
		F20	C_13_H_11_BrN_2_O_2_	307.0077	307.0076	–0.33	
		F4	C_8_H_3_BrN_2_O	222.9502	222.9502	0.00	
		F21	C_8_H_5_O	85.0648	85.0647	–1.18	
M5b	Aliphatic hydroxylation in pentylic chain + Alcohol oxidation into ketone or aldehyde	-	C_20_H_26_BrN_3_O_4_	452.1180	452.1180	0.00	15.61
		F19	C_18_H_22_BrN_3_O_2_	392.0968	392.0970	0.51	
		F20	C_13_H_11_BrN_2_O_2_	307.0077	307.0076	–0.33	
		F4	C_8_H_3_BrN_2_O	222.9502	222.9511	4.04	
		F21	C_8_H_5_O	85.0648	85.0647	–1.18	
M5c	Aliphatic hydroxylation in pentylic chain + Alcohol oxidation into ketone or aldehyde	-	C_20_H_26_BrN_3_O_4_	452.1180	452.1179	0.22	15.75
		F19	C_18_H_22_BrN_3_O_2_	392.0968	392.0967	–0.26	
		F20	C_13_H_11_BrN_2_O_2_	307.0077	307.0079	0.65	
		F4	C_8_H_3_BrN_2_O	222.9502	222.9503	0.45	
		F21	C_8_H_5_O	85.0648	85.0647	–1.18	
M6a	Aliphatic hydroxylation in *tert*-butyl moiety + Alcohol oxidation into aldehyde	-	C_20_H_26_BrN_3_O_4_	452.1180	452.1181	0.22	16.13
		F22	C_18_H_22_BrN_3_O_2_	392.0968	392.0966	–0.51	
		F11	C_17_H_22_BrN_3_O	364.1019	364.1015	–1.10	
		F3	C_13_H_13_BrN_2_O	293.0284	293.0283	–0.34	
		F4	C_8_H_3_BrN_2_O	222.9502	222.9501	–0.45	
M6b	Aliphatic hydroxylation in *tert*-butyl moiety + Alcohol oxidation into aldehyde	-	C_20_H_26_BrN_3_O_4_	452.1180	452.1180	0.00	16.31
		F22	C_18_H_22_BrN_3_O_2_	392.0968	392.0970	0.51	
		F11	C_17_H_22_BrN_3_O	364.1019	364.1026	1.92	
		F3	C_13_H_13_BrN_2_O	293.0284	293.0283	–0.34	
		F4	C_8_H_3_BrN_2_O	222.9502	222.9500	–0.90	
M6c	Aliphatic hydroxylation in *tert*-butyl moiety + Alcohol oxidation into aldehyde	-	C_20_H_26_BrN_3_O_4_	452.1180	452.1180	0.00	16.55
		F22	C_18_H_22_BrN_3_O_2_	392.0968	392.0961	–1.79	
		F11	C_17_H_22_BrN_3_O	364.1019	364.1040	5.77	
		F3	C_13_H_13_BrN_2_O	293.0284	293.0284	0.00	
		F4	C_8_H_3_BrN_2_O	222.9502	222.9502	0.00	
M7	Aliphatic hydroxylation in pentylic chain + Alcohol oxidation into carboxylic acid	-	C_20_H_26_BrN_3_O_5_	468.1129	468.1133	0.85	14.85
		*F*23	C_19_H_22_BrN_3_O_3_	436.0867	436.0875	1.83	
		F24	C_18_H_22_BrN_3_O_3_	408.0917	408.0917	0.00	
		F25	C_13_H_11_BrN2O_3_	323.0026	323.0026	0.00	
		F4	C_8_H_3_BrN_2_O	222.9502	222.9503	0.45	
		F26	C_5_H_8_O_2_	101.0597	101.0596	–0.99	
M8	Aliphatic hydroxylation in *tert*-butyl moiety + Alcohol oxidation into carboxylic acid	-	C_20_H_26_BrN_3_O_5_	468.1129	468.1129	0.00	16.16
		F27	C_19_H_24_BrN_3_O_3_	422.1074	422.1073	–0.24	
		F28	C_18_H_22_BrN_3_O_3_	408.0917	408.0919	0.49	
		F3	C_13_H_13_BrN_2_O	293.0284	293.0284	0.00	
		F4	C_8_H_3_BrN_2_O	222.9502	222.9501	–0.45	
M9	Aliphatic hydroxylation in pentylic chain + Aliphatic hydroxylation in *tert*-butyl moiety + Tert-butylic alcohol oxidation into carboxylic acid	-	C_20_H_26_BrN_3_O_6_	484.1078	484.1078	0.00	13.91
		F29	C_20_H_24_BrN_3_O_5_	466.0972	466.0960	–2.57	
		F30	C_19_H_24_BrN_3_O_4_	438.1023	438.1024	0.23	
		F18	C_17_H_22_BrN_3_O_2_	380.0968	380.0975	1.84	
		F8	C_13_H_13_BrN_2_O_2_	309.0233	309.0240	2.27	
		F4	C_8_H_3_BrN_2_O	222.9502	222.9502	0.00	
		F5	C_8_H_5_	69.0699	69.0700	1.45	
M10	Aliphatic hydroxylation in pentylic chain + Aliphatic hydroxylation in *tert*-butyl moiety + Pentylic alcohol oxidation into ketone or aldehyde + Tert-butylic alcohol oxidation into carboxylic acid	-	C_20_H_24_BrN_3_O_6_	482.0921	482.0921	0.00	13.82
		F31	C_17_H_20_BrN_3_O_2_	436.0867	436.0869	4.46	
		F32	C_17_H_20_BrN_3_O_2_	378.0812	378.0798	–3.70	
		F21	C_13_H_11_BrN_2_O_2_	307.0077	307.0078	0.33	
		F4	C_8_H_3_BrN_2_O	222.9502	222.9503	0.45	
		F22	C_5_H_8_O	85.0648	85.0648	0.00	
M11	Ester hydrolysis	-	C_19_H_26_BrN_3_O_3_	424.1230	424.1231	0.24	16.95
		F2	C_18_H_24_BrN_3_O	378.1176	378.1175	–0.26	
		F3	C_13_H_13_BrN_2_O	293.0284	293.0283	–0.34	
		F4	C_8_H_3_BrN_2_O	222.9502	222.9502	0.00	
		F33	C_5_H_11_N	86.0964	86.0963	–1.16	
M12	Ester hydrolysis + Aliphatic hydroxylation in pentylic chain	-	C_19_H_26_BrN_3_O_4_	440.1180	440.1179	–0.23	14.75
		F7	C_18_H_24_BrN_3_O_3_	394.1125	394.1123	–0.51	
		F8	C_13_H_13_BrN_2_O_2_	309.0233	309.0247	4.53	
		F4	C_8_H_3_BrN_2_O	222.9502	222.9506	1.79	
		F33	C_5_H_11_N	86.0964	86.0964	0.00	
		F5	C_5_H_8_	69.0699	69.0699	0.00	
M13	Ester hydrolysis + Aliphatic hydroxylation in pentylic chain + Aliphatic hydroxylation in *tert*-butyl moiety	-	C_19_H_26_BrN_3_O_5_	456.1129	456.1131	0.44	13.04
		F16	C_19_H_24_BrN_3_O_4_	438.1023	438.0998	–5.70	
		F17	C_19_H_22_BrN_3_O_3_	420.0917	420.0916	–0.24	
		F18	C_17_H_22_BrN_3_O_2_	380.0968	380.0966	–0.53	
		F8	C_13_H_13_BrN_2_O_2_	309.0233	309.0237	1.29	
		F4	C_8_H_3_BrN_2_O	222.9502	222.9503	0.45	
		F5	C_5_H_8_	69.0699	69.0699	0.00	
M14	Ester hydrolysis + Aliphatic hydroxylation in *tert*-butyl moiety + Tert-butylic alcohol oxidation into aldehyde	-	C_19_H_24_BrN_3_O_4_	438.1023	438.1022	–0.23	15.87
		F22	C_18_H_22_BrN_3_O_2_	392.0968	392.0956	–3.06	
		F11	C_17_H_22_BrN_3_O	364.1019	364.1038	5.22	
		F3	C_13_H_13_BrN_2_O	293.0284	293.0281	–1.02	
		F4	C_8_H_3_BrN_2_O	222.9502	222.9499	–1.35	
M15	Ester hydrolysis + Aliphatic hydroxylation in *tert*-butyl moiety + Aliphatic hydroxylation in pentylic chain + Pentylic alcohol oxidation into ketone or aldehyde	-	C_19_H_24_BrN_3_O_5_	454.0972	454.0974	0.44	12.92
		F31	C_17_H_20_BrN_3_O_2_	436.0867	436.0853	–3.21	
		F32	C_17_H_20_BrN_3_O_2_	378.0812	378.0812	0.00	
		F21	C_13_H_11_BrN_2_O_2_	307.0077	307.0078	0.33	
		F22	C_5_H_8_O	85.0648	85.0648	0.00	
M16	Ester hydrolysis + Aliphatic hydroxylation in pentylic chain + Aliphatic hydroxylation in *tert*-butyl moiety + Tert-butylic alcohol oxidation into aldehyde	-	C_19_H_24_BrN_3_O_5_	454.0972	454.0973	0.22	13.34
		F34	C_17_H_20_BrN_3_O_2_	436.0867	436.0893	5.96	
		F35	C_19_H_22_BrN_3_O_3_	408.0917	408.0922	1.23	
		F18	C_17_H_22_BrN_3_O_2_	380.0968	380.0964	–1.05	
		F8	C_13_H_13_BrN_2_O_2_	309.0233	309.0232	–0.32	
		F4	C_8_H_3_BrN_2_O	222.9502	222.9501	–0.45	
		F5	C_5_H_8_	69.0699	69.0700	1.45	
M17	Ester hydrolysis + Aliphatic hydroxylation in pentylic chain + Aliphatic hydroxylation in *tert*-butyl moiety + Lactone formation	-	C_19_H_24_BrN_3_O_4_	438.1023	438.1023	0.00	13.67
		F36	C_19_H_22_BrN_3_O_3_	420.0917	420.0919	0.48	
		F18	C_17_H_22_BrN_3_O_2_	380.0968	380.0979	2.89	
		F8	C_13_H_13_BrN_2_O_2_	309.0233	309.0242	2.91	
		F4	C_8_H_3_BrN_2_O	222.9502	222.9501	–0.45	
		F37	C_6_H_8_O_2_	113.0597	113.0602	4.42	
		F5	C_5_H_8_	69.0699	69.0699	0.00	
M18a	Ester hydrolysis + Aliphatic hydroxylation in pentylic chain + Aliphatic hydroxylation in *tert*-butyl moiety + Pentylic alcohol oxidated into ketone or aldehyde + Lactone formation	-	C_19_H_22_BrN_3_O_4_	436.0869	436.0867	–0.46	13.54
		F32	C_17_H_20_BrN_3_O_2_	378.0812	378.0800	–3.17	
		F21	C_13_H_11_BrN_2_O_2_	307.0077	307.0076	–0.33	
		F4	C_8_H_3_BrN_2_O	222.9502	222.9512	4.48	
		F22	C_5_H_8_O	85.0648	85.0647	–1.18	
M18b	Ester hydrolysis + Aliphatic hydroxylation in pentylic chain + Aliphatic hydroxylation in *tert*-butyl moiety + Pentylic alcohol oxidated into ketone or aldehyde + Lactone formation	-	C_19_H_22_BrN_3_O_4_	436.0869	436.0870	0.23	13.76
		F32	C_17_H_20_BrN_3_O_2_	378.0812	378.0829	4.50	
		F21	C_13_H_11_BrN_2_O_2_	307.0077	307.0076	–0.42	
		F22	C_5_H_8_O	85.0648	85.0647	–1.18	
M19	Ester hydrolysis + Aliphatic hydroxylation in *tert*-butyl moiety + Lactone formation + Loss of pentylic chain	-	C_14_H_14_BrN_3_O_3_	352.0291	352.0291	0.00	12.70
		F38	C_13_H_14_BrN_3_O_2_	324.0342	324.0350	2.47	
		F39	C_12_H_12_BrN_3_O	294.0237	294.0239	0.68	
		F4	C_8_H_3_BrN_2_O	222.9502	222.9502	0.00	

The metabolic reactions observed *in vitro* were: aliphatic hydroxylation in pentylic chain, aliphatic hydroxylation in *tert*-butyl moiety, aromatic hydroxylation, alcohol oxidation into ketone or aldehyde, alcohol oxidation into carboxylic acid, ester hydrolysis, lactone formation, and loss of pentylic chain.

MS^2^-based MN was employed as an exploratory and supportive tool to guide the identification of MDMB-5′Br-PINACA metabolites (Figure 3). The molecular network revealed a tight clustering of the parent compound and related features, reflecting high spectral similarity and close structural relationships. The node corresponding to MDMB-5′Br-PINACA was used as a reference to systematically search for connected nodes (primary nodes) exhibiting similar MS^2^ fragmentation patterns, which were considered as candidates for structurally related metabolites. Additionally, further nodes that were linked to the primary nodes were also considered. In total, 34 nodes were presented in this network. Among these, 27 represented features associated as potential MDMB-5′Br-PINACA metabolites, being presented only in metabolism samples, while 7 connected features were also detected in negative controls, being excluded from a metabolic origin. From these nonmetabolic features, five were associated with dehydrogenation in the pentylic chain (C_20_H_26_BrN_3_O_3_, all mass errors less than 1 ppm), and the other two associated with a loss of the pentylic chain (C_15_H_18_BrN_3_O_3_, *m*/*z* 368.06046, Δ = 0.27 ppm), and a loss of the pentylic chain followed by the loss of the bromine atom (C_15_H_19_N_3_O_3_, *m*/*z* 290.1499, Δ = 0.00 ppm). The remaining 27 features were also structurally elucidated and confidently assigned as MDMB-5′Br-PINACA metabolites, enabling construction of a comprehensive phase I metabolic scheme.

**Figure 3:**
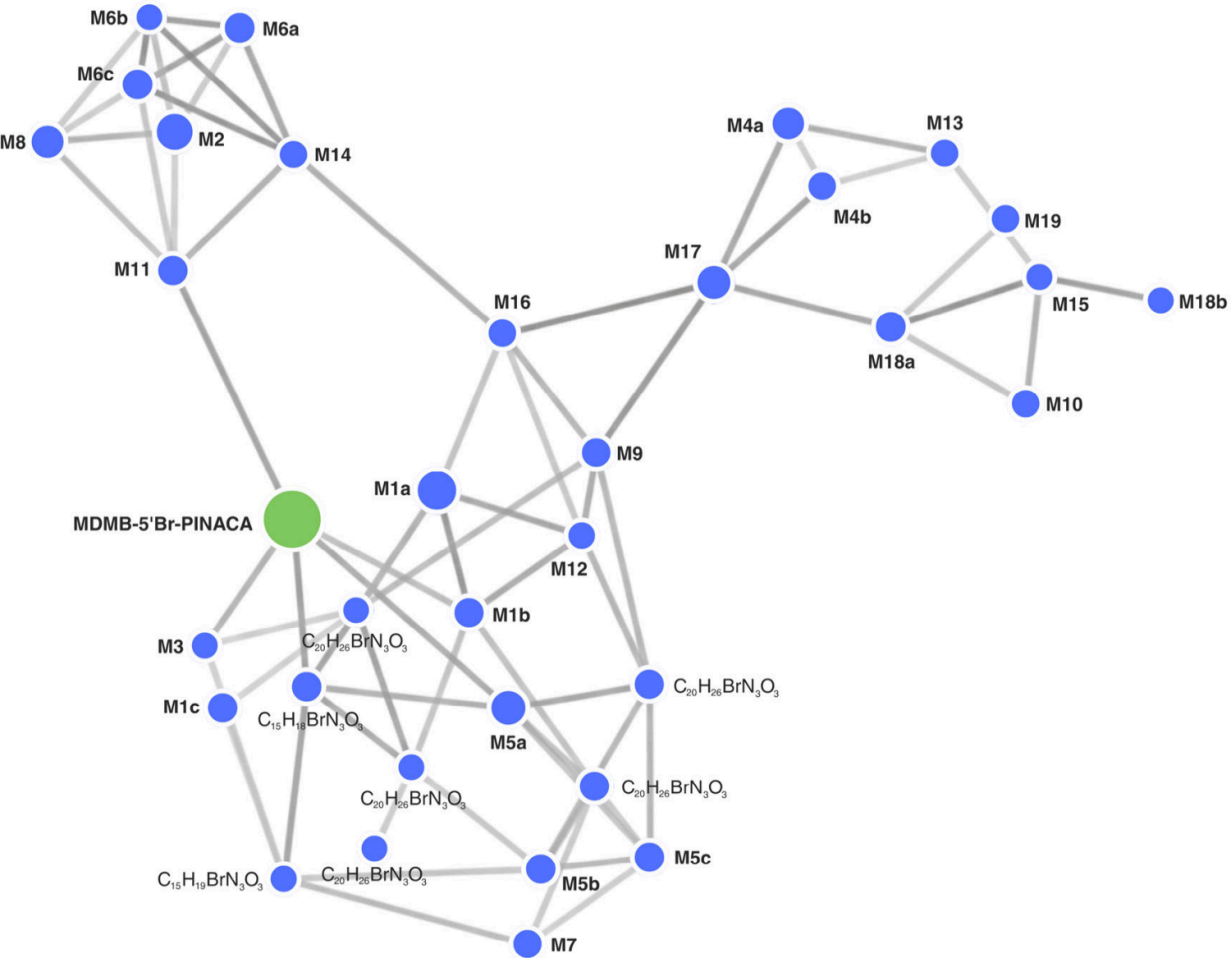
MS^2^-based molecular network of MDMB-5′Br-PINACA metabolites generated from LC-HRMS data after incubation in pooled human liver microsomes (pHLM). The network comprises 34 nodes, including 27 metabolites detected exclusively in incubation samples and 7 features also present in negative controls. The cosine similarity threshold was set to 0.4. Node size is proportional to feature intensity (peak area), and edge thickness reflects the cosine similarity score between MS^2^ fragmentation spectra, with darker edges indicating higher spectral similarity.

Three monohydroxylated metabolites were identified, differing exclusively in the site of oxidation and chromatographic characteristics. Metabolites 1a, 1b, and 1c (M1a, M1b, and M1c) were assigned to aliphatic hydroxylation of the pentyl side chain, eluting at 15.91, 15.98, and 16.09 min, respectively. These metabolites exhibited identical precursor ions and highly similar MS^2^ fragmentation patterns, consistent with positional isomers arising from hydroxylation at different carbon atoms along the pentyl chain. However, due to the absence of authentic reference standards and the resulting inability to confirm retention time matching, the exact position of hydroxylation could not be unequivocally established. Previous metabolic studies on synthetic cannabinoids with a pentyl side chain, such as JWH-018 and AM-2201, frequently report monohydroxylation at the C4- and C5-positions as predominant pathways in human and *in vitro* systems.^40,41^ These findings suggest a tendency for CYP-mediated oxidation to occur at the ω-1 (penultimate) and ω (terminal) positions of the aliphatic chain.^7^ Comparative MS^2^ analysis relative to the parent MDMB-5′Br-PINACA demonstrated preservation of the indazole core and *tert*-butyl fragments, while diagnostic ions such as F4, F5, F6, F7 and F8 confirmed oxidation localized to the aliphatic side chain. Percentage relative area of these three signals demonstrated that M1a was preferentially formed, reaching 8.25% of metabolites amount after 30 min of incubation, being the fourth major metabolite in this time. On the other hand, M1b and M1c reached only 3.00 and 1.89%, respectively. After 60 min, the proportion of these metabolites remained similar, showing 5.84% for M1a, 2.13% for M1b, and 1.25% for M1c. Metabolite 2 (M2) was characterized as a *tert*-butyl-hydroxylated derivative with a retention time of 16.23 min. MS^2^ spectrum analysis showed a diagnostic fragment ion (F11) consistent with oxidation at the *tert*-butyl moiety, while fragments associated with the pentyl chain and indazole core remained unchanged. M2 was found as the second most abundant metabolite in pHLM medium after 30 min of incubation, reaching 11.10% of the metabolites total area. Metabolite 3 (M3), eluting at 17.28 min, was identified as an aromatic hydroxylation product, with MS^2^ spectral features indicating modification within the indazole aromatic system, supported by fragment ion F15. From the three hydroxylation regions, the aromatic hydroxylation was the less prominent, reaching 1.50 and 0.99%, after 30 and 60 min, respectively. Further hydroxylation reactions were also detected in pHLM medium, resulting in dihydroxylated metabolites. Metabolites 4a (M4a) and 4b (M4b), eluting at 13.85 and 14.27 min, respectively, were produced by successive phase I oxidation reactions involving hydroxylation of the pentyl side chain and the *tert*-butyl moiety. Although the exact positions of hydroxylation could not be conclusively assigned through LC-HRMS, MS^2^ fragmentation supported the presence of two hydroxyl groups through diagnostic fragment ions F16, F17, and F18. Low amounts of these two metabolites were detected in our study. While M4a reached 2.04% in 30 min and dropped to 1.52% after 60 min, M4b was only detected in the first incubation time (0.51%). Hydroxylation reactions were identified as one of the most important metabolic reactions of MDMB-5′Br-PINACA in the pHLM system, reflecting the high susceptibility of this compound, as well as other SC, to CYP450-mediated oxidation.^7,42^ Notably, both BioTransformer 3.0 and XenoSite consistently predicted these reactions in all the three molecular sites (pentyl chain, *tert*-butyl moiety, and indazole ring).

Building upon the primary mono- and dihydroxylated, additional metabolites were generated through secondary phase I oxidation reactions involving the conversion of hydroxyl groups into carbonyl functionalities. Metabolites 5a, 5b, and 5c (M5a, M5b, and M5c) resulted from aliphatic hydroxylation of the pentyl chain followed by oxidation of the introduced hydroxyl group to a carbonyl moiety, yielding either ketone or aldehyde derivatives depending on the oxidation site. Similarly to M1a-M1c and M4a and M4b, these metabolites represent positional isomers with highly similar MS^2^ fragmentation patterns, the exact sites of biotransformation could not be unambiguously determined in the absence of reference standards. These modifications were assigned using diagnostic fragments F19 and F20, which characterize the carbonyl insertion in the five-carbon tail. Interestingly, M5a represented the most prevalent metabolite under the experimental conditions after 30 min of incubation (19.16%), maintaining its high percentage levels in the following time (15.82%). However, M5b and M5c were less produced, not exceeding 5% of the metabolites production. The same reactions were also detected but at a different molecular site. Metabolites 6a, 6b, and 6c (M6a, M6b, and M6c) were generated by *tert*-butyl hydroxylation followed by further oxidation to aldehyde-containing products. MS^2^ fragmentation spectra for these metabolites were consistent with alcohol-to-carbonyl conversion, as reflected by characteristic diagnostic fragment ion, F22 – even though the exact sites of biotransformation could not be determined. These three potential isomers did not exceed 4% of the total metabolite formation. Further oxidation of hydroxylated intermediates led to the formation of carboxylic acid derivatives. Following initial hydroxylation, several metabolites underwent secondary oxidation of the introduced hydroxyl groups, leading to the formation of carbonyl-containing metabolites. These transformations are characteristic of synthetic cannabinoid metabolism and represent a logical progression from primary alcohol intermediates already described to similar SC such as ADB-BUTINACA, BZO-POXIZID, ADB-P-5′Br-INACA and others.^43,44^ BioTransformer 3.0 extensively predicted these sequential oxidative transformations, reflecting its rule-based framework designed to capture multistep metabolic cascades. XenoSite similarly indicated high probabilities for stable and unstable oxidation in this region, although without explicitly enumerating downstream metabolites.

Metabolite 7 (M7) originated from following oxidation reactions producing carboxylic acid in the pentyl side chain, whereas metabolite 8 (M8) resulted from analogous oxidation within the *tert*-butyl moiety. MS^2^ spectra supported carboxylic acid formation through fragment ions characteristic of terminal oxidation (F24 and F25), while conservation of indazole-related fragments confirmed localized modification (F4). Both carboxylic acid derivatives showed increasing formation over time, with M7 reaching 5.01% and M8 20.35% - being the most abundant metabolite of MDMB-5′Br-PINACA formed after 60 min. Metabolite 9 (M9) was identified as a multistep oxidation product, formed through two successive hydroxylations (pentyl side chain and *tert*-butyl group), followed by an oxidation of the *tert*-butyl-derived primary alcohol to a carboxylic acid. These reactions were characterized through fragments F8, demonstrating the aliphatic hydroxylation in the pentyl tail, F18 indicating the oxidation in both pentylic tail and *tert*-butyl moiety, and F29 and F30 pointing to a carboxylic acid in *tert*-butylic region. Similarly, metabolite 10 (M10) was generated by hydroxylation at both regions, followed by dual oxidation reactions, yielding a carbonyl group on the pentyl chain and on the *tert*-butyl moiety, as supported by diagnostic MS^2^ fragments (F31 and F32). M9 was found in greater amounts after 60 min (5.09%), while M10 was only identified in 30 min incubation samples (2.07%). Therefore, the formation of carboxylic acid-containing metabolites represents advanced oxidative metabolism being already demonstrated to other SC such as MDMB-4en-PINACA.^45^ These findings are in strong agreement with BioTransformer 3.0 predictions, which proposed extensive multistep oxidation culminating in carboxylic acid formation. Although XenoSite does not explicitly predict final metabolite structures, it assigned high oxidation probabilities to the same molecular regions, indirectly supporting the formation of these highly oxidized products.

Metabolite 11 (M11) was identified as a carboxylic acid derivative formed by hydrolysis of the terminal methyl ester of MDMB-5′Br-PINACA. Its precursor ion exhibited a mass decrease of 14.0157 Da (CH_2_) relative to the parent compound, consistent with the loss of the methyl group associated with the ester functionality, while fragments corresponding to the indazole core, pentyl side chain, and *tert*-butyl moiety remained preserved (F2, F3, and F4). M11 was found as the fourth most abundant metabolite in pHLM after 60 min, reaching 6.51%. Metabolite 12 (M12) was characterized as a monohydroxylated derivative of M11 occurring at the pentyl side chain, with fragments F7 and F8 supporting this substitution. Moreover, metabolite 13 (M13) was identified as a dihydroxylated metabolite derived from M11, with fragmentation patterns consistent with hydroxyl group incorporation at both pentyl and *tert*-butyl regions (F8, F16, F17, and F18). The hydroxylated derivatives of the hydrolyzed metabolite were found less abundant than M11, not exceeding 3%. Additionally, metabolite 14 (M14) was tentatively identified as an aldehyde derivative arising from further oxidation of a hydroxylated *tert*-butyl moiety following ester hydrolysis. Characteristic fragment ions associated with the indazole core (F3 and F4) indicated that this structural motif remained unaltered. Fragment ion F11 suggested metabolic modification within the *tert*-butyl region, whereas fragment ion F22 was consistent with the presence of a terminal carbonyl functionality. Although the corresponding monohydroxylated *tert*-butyl intermediate was not detected, the observed fragmentation pattern supports a sequential metabolic pathway involving ester hydrolysis, *tert*-butyl hydroxylation, and subsequent oxidation to an aldehyde. M14 represented a minor biotransformation product, accounting for less than 2% of the total metabolite area. Metabolites 15 (M15) and 16 (M16) were identified as products of alcohol oxidation in M13 producing carbonyl functionalities. M15 was characterized by the oxidation reaction occurring at the five-carbon aliphatic side chain, yielding either a ketone or aldehyde functionality depending on the position of the hydroxyl group. The MS^2^ spectrum of M15 displayed diagnostic fragment ions F31 and F32, which are characteristic of carbonyl insertion, together with fragment F21, supporting localization of the carbonyl group within the pentyl chain. In contrast, M16 exhibited this same oxidation in *tert*-butyl moiety, leading to the formation of an aldehyde functionality. Diagnostic MS^2^ fragment ions F8 and F18 supported the presence of hydroxylation at the pentyl side chain, while fragment F35 was indicative of both hydroxylation and subsequent carbonyl formation within the *tert*-butyl region. While M15 was only detected after 60 min (1.01%), M16 was found in both incubation times, even though its abundance did not exceed 2%. Hydrolysis of the methyl ester constituted a major and mechanistically favorable metabolic pathway of methyl ester derivative-SC, yielding carboxylic acid derivative metabolites.^7,43,45^ Moreover, this reaction was strongly predicted by XenoSite, which identified the ester bond as a highly labile site, whereas BioTransformer 3.0 placed less emphasis on this transformation. Subsequent hydroxylation and oxidation of the hydrolyzed metabolites, demonstrate that ester cleavage does not terminate metabolic processing but instead generates intermediates susceptible to further phase I reactions. Previous studies on synthetic cannabinoids have similarly reported extensive secondary metabolism following ester hydrolysis, including hydroxylation and alcohol oxidation to aldehydes, ketones, and carboxylic acids.

Metabolite 17 (M17) was formed through intramolecular lactone formation of M13. This cyclization was proposed to occur via nucleophilic attack of the hydroxyl group introduced at the *tert*-butyl moiety onto the carboxylic acid carbonyl, resulting in lactone ring closure with concomitant loss of a water molecule.^46^ Similar reactions were previously reported to other SC, such as ADB-CHMINACA in human hepatocytes and ABM-CHIMICA in rats’ urine, liver, and kidney.^47,48^ The MS^2^ fragmentation pattern supported this rearrangement through diagnostic ions F36 and F37 - consistent with ring formation. M17 presented an increasing formation over the incubation times, reaching 5.07% after 60 min. Metabolites 18a (M18a) and 18b (M18b) were identified as closely related lactone-forming metabolites derived from M17. Subsequent oxidation of the hydroxyl group introduced in the pentyl chain yielded a carbonyl functionality. These two metabolites were differentiated by their retention times, with M18a eluting at 13.54 and M18b at 13.76 min, even though the precise position of metabolism was not determined in the absence of reference standards or NMR analysis. These two metabolites may represent positional isomers differing in the site of pentyl-chain hydroxylation prior to oxidation. M18a was detected in both incubation times, reaching 3.57% after 60 min, and M18b was only identified after 30 min, showing an abundance of 0.49%. A third feature tentatively assigned as metabolite 18c was observed at a retention time of 13.85 min. However, due to its low signal intensity, no MS^2^ fragmentation spectrum was acquired. Therefore, this feature was not reported as a MDMB-5′Br-PINACA metabolite. Finally, metabolite 19 (M19) was also characterized as lactone-derivative, formed by a *N*-dealkylation of M17 in the five-carbon tail. Subsequent fragmentation patterns further indicated loss of the pentyl side chain, yielding a truncated lactone-containing structure, as supported by the absence of characteristic pentyl-associated fragment ions and preservation of fragments F38 and F39, supporting the lactone presence. Lactone intramolecular formation was not predicted by either *in silico* platform, likely reflecting intrinsic limitations of current models, which are primarily trained on enzyme-mediated reactions (mainly CYP450-driven transformations) and optimized for common pathways such as hydroxylation and oxidation. In contrast, lactone formation involves more complex, structure-dependent intramolecular rearrangements, typically requiring intermediate functional group formation followed by cyclization. Such transformations are underrepresented in training data sets and are not readily captured by rule-based or machine learning approaches, highlighting a key limitation of current *in silico* tools in predicting nonlinear metabolic pathways, particularly for structurally new synthetic cannabinoids. However, *N*-dealkylation leading to loss of the pentyl side chain was predicted by XenoSite, which identified high-probability hotspots at nitrogen-containing regions of the molecule. The experimental confirmation of this pathway supports the predictive value of XenoSite for nonoxidative clearance mechanisms and complements the oxidative focus of BioTransformer 3.0. Based on the phase I metabolites identified herein, it was also possible to purpose a metabolic pathway for MDMB-5′Br-PINACA in pHLM (Figure 4) and the relative formation ratio of each metabolite after 30 and 60 min of incubation (Figure 5).

**Figure 4:**
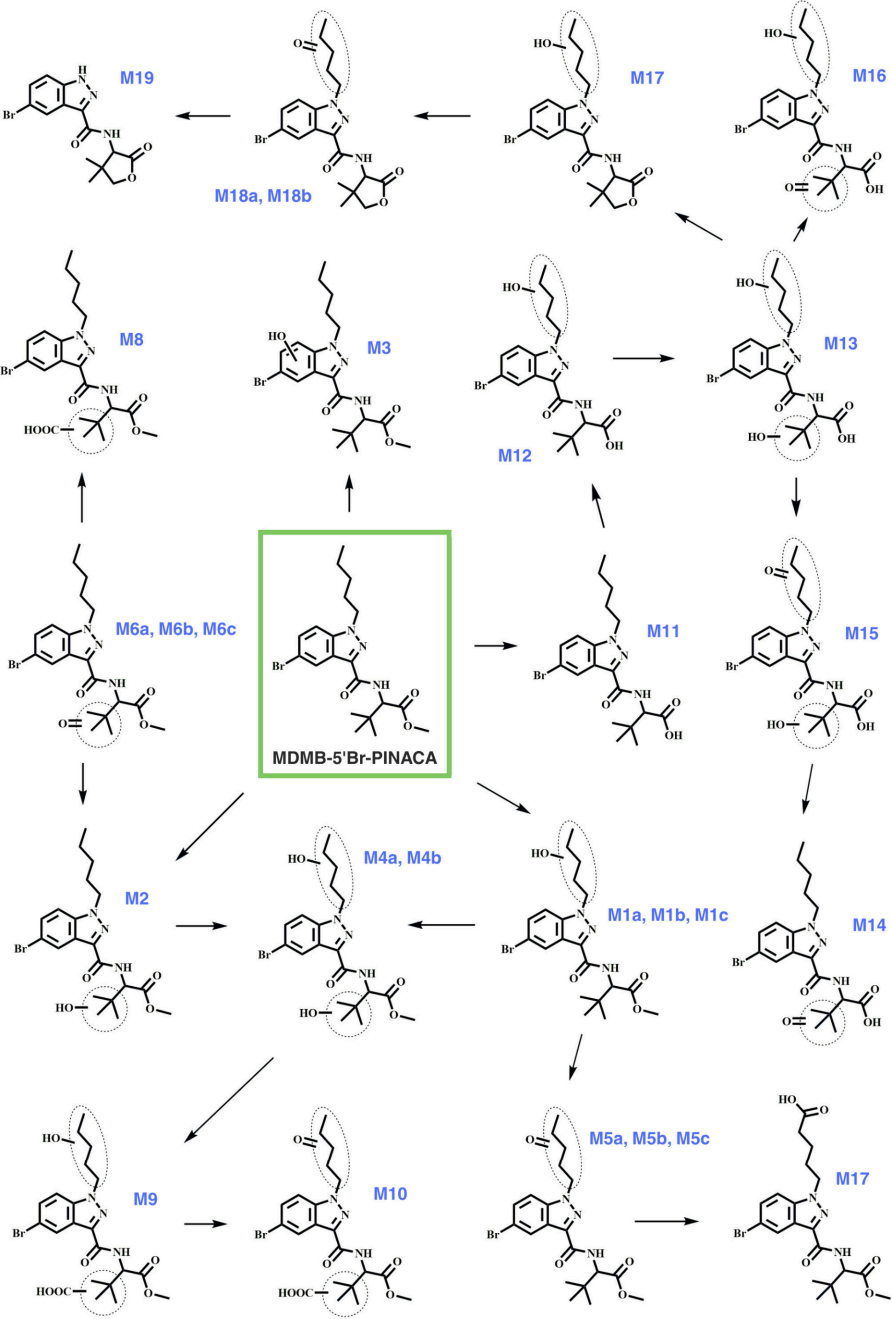
Metabolic pathway of MDMB-5′Br-PINACA in pooled human liver microsomes (pHLM).

**Figure 5:**
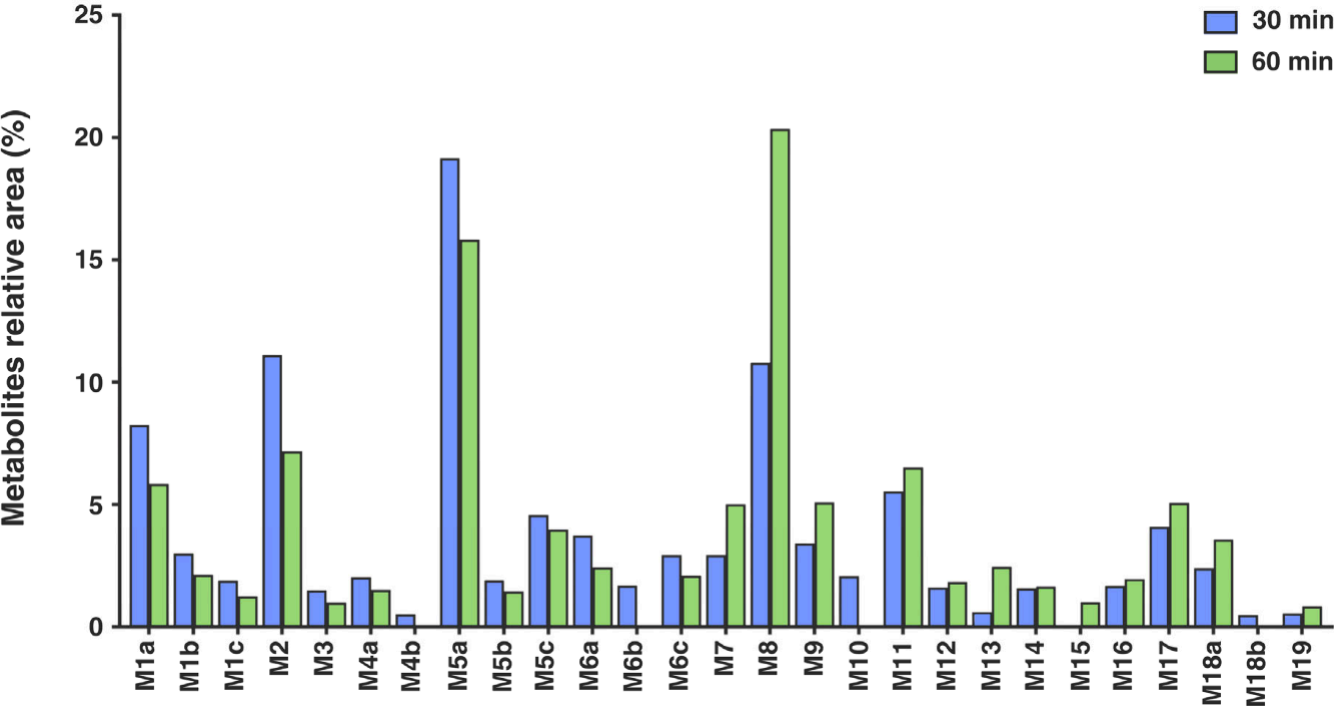
Percentage of MDMB-5′Br-PINACA metabolites relative area produced in pooled human liver microsomes (pHLM) after 30 (blue) and 60 (green) minutes.

From a toxicological perspective, the extensive oxidative metabolism observed for MDMB-5′Br-PINACA suggests high metabolic liability, which may influence both duration of action and formation of potentially active or reactive intermediates. Hydroxylated metabolites, as reported for other synthetic cannabinoids, may retain affinity for CB_1_R, thereby contributing to pharmacological effects.^41,49^ Additionally, the formation of oxidized species, including aldehydes and potential epoxide precursors, raises the possibility of reactive intermediates involved in toxicity.^50^ The identification of carboxylic acid derivatives and intramolecular lactone formation highlights structural transformations that may impact both biological persistence and physicochemical properties relevant for excretion. Importantly, metabolites M5a, M8, and M2 may also represent suitable analytical targets for toxicological screening, particularly in cases where the parent compound is no longer detectable. Based on the semiquantitative LC-HRMS data obtained, these metabolites were consistently observed at relatively higher signal intensities and remained detectable over time, indicating both relevant formation and metabolic stability under the experimental conditions. In particular, M5a and M2 correspond to common phase I biotransformation products typically associated with synthetic cannabinoids, while M8, a more extensively oxidized metabolite, presents increased polarity that may further favor urinary excretion and detection. Together, these features support their applicability as robust analytical targets in urine-based screening strategies.

Although further *in vivo* validation is required to confirm the relevance of these metabolites, the present findings provide important preliminary insights into the metabolic profile of this emerging brominated synthetic cannabinoid. The use of pHLM as a controlled and widely accepted *in vitro* model enabled the characterization of CYP450-mediated phase I reactions, which is particularly valuable for newly identified substances lacking prior pharmacological or toxicological data. Future studies involving *in vivo* models or authentic biological samples will be essential to establish the translational relevance of these metabolic pathways. Moreover, the use of a single incubation experiment represents a limitation of this study, which may impact robust quantitative or semiquantitative assessment. Accordingly, relative metabolite abundances should be interpreted as exploratory. However, as the primary objective was qualitative metabolite profiling and structural elucidation, this limitation does not substantially affect the validity or relevance of the metabolic pathways here described.

## 4 Conclusions

This study provides a comprehensive characterization of the phase I metabolism of MDMB-5′Br-PINACA using an integrated *in silico* and *in vitro* strategy supported by LC-HRMS and MN. Twenty-seven metabolites were annotated with level 2 of confidence, highlighting an extensive metabolic liability of this SC, with oxidative transformations of the pentyl side chain and *tert*-butyl moiety representing the predominant pathways. Importantly, ester hydrolysis, secondary alcohol oxidations, intramolecular lactone formation, and *N*-dealkylation with loss of the pentyl chain further expand the metabolic landscape, generating structurally diverse metabolites that may serve as potential analytical targets for toxicological screening, although their *in vivo* persistence and toxicological relevance require further investigation. Comparison between computational predictions and experimental findings demonstrated substantial concordance for primary oxidative reactions, while also revealing metabolic routes not anticipated by *in silico* models alone, emphasizing the indispensability of experimental validation. MN proved to be a powerful auxiliary tool, enabling efficient prioritization of metabolite-related features, visualization of structural relationships, and improved confidence in metabolite annotation. From a forensic and clinical toxicology perspective, the metabolites identified in this study may represent relevant analytical targets for the detection of MDMB-5′Br-PINACA exposure, particularly in cases where the parent compound is no longer detectable and may also contribute to the overall toxicological profile of this compound. More broadly, this work illustrates how the combined application of *in silico* prediction, *in vitro* pHLM studies, and MN can substantially enhance metabolite elucidation and should be considered a robust framework for the investigation of emerging synthetic cannabinoids and other new psychoactive substances.

Overall, these findings support ongoing toxicological surveillance of emerging halogenated synthetic cannabinoids and highlight the importance of further studies addressing the biological activity and toxicological relevance of their metabolites, while reinforcing the value of combining *in silico* predictions with *in vitro* metabolism approaches for the rapid characterization of newly identified psychoactive substances.

## Supplementary Material



## Data Availability

All data presented in this study are available in this article.

## Notes

MS^1^-mass spectra of MDMB-5′Br-PINACA metabolites considering phase I metabolism in pooled human liver microsomes (pHLM) by LC-HRMS using electrospray ionization in positive mode (ESI^+^); MS^2^-mass spectra of MDMB-5′Br-PINACA metabolites considering phase I metabolism in pooled human liver microsomes (pHLM) by LC-HRMS using electrospray ionization in positive mode (ESI^+^ (PDF)

## References

[1] Zawilska J. B., Andrzejczak D. (2015). Next Generation of Novel Psychoactive Substances on
the Horizon - A Complex Problem to Face. Drug
Alcohol Depend.

[2] Tettey, J. N. A. ; Crean, C. ; Ifeagwu, S. C. ; Raithelhuber, M. Emergence, Diversity, and Control of New Psychoactive Substances: A Global Perspective. In New Psychoactive Substances: Pharmacology, Clinical, Forensic and Analytical Toxicology; Maurer, H. H. , Brandt, S. D. , Eds.; Springer International Publishing: Cham, 2018; pp 51–67. 10.1007/164_2018_127. 29896655

[3] Potts A. J., Cano C., Thomas S. H. L., Hill S. L. (2020). Synthetic Cannabinoid
Receptor Agonists: Classification and Nomenclature. Clin Toxicol (Phila).

[4] European Monitoring Centre for Drugs and Drug Addiction. European Drug Report 2024: Trends and Developments. https://www.emcdda.europa.eu/publications/european-drug-report/2024_en (accessed 2026-01-03).

[5] Rodrigues T.
B., Souza M. P., de Melo Barbosa L., de Carvalho Ponce J., Júnior L. F. N., Yonamine M., Costa J. L. (2022). Synthetic Cannabinoid
Receptor Agonists Profile in Infused Papers Seized in Brazilian Prisons. Forensic Toxicol.

[6] Fantegrossi W. E., Moran J. H., Radominska-Pandya A., Prather P. L. (2014). Distinct Pharmacology
and Metabolism of K2 Synthetic Cannabinoids Compared to Δ(9)-THC:
Mechanism Underlying Greater Toxicity?. Life
Sci..

[7] Diao, X. ; Huestis, M. A. New Synthetic Cannabinoids Metabolism and Strategies to Best Identify Optimal Marker Metabolites. Front. Chem. 2019, 7. 10.3389/fchem.2019.00109. PMC6409358 30886845

[8] Atwood B. K., Lee D., Straiker A., Widlanski T. S., Mackie K. (2011). CP47,497-C8 and JWH073,
Commonly Found in “Spice” Herbal Blends, Are Potent
and Efficacious CB(1) Cannabinoid Receptor Agonists. Eur. J. Pharmacol..

[9] Andrews R., Jorge R., Christie R., Gallegos A. (2023). From JWH-018 to OXIZIDS:
Structural Evolution of Synthetic Cannabinoids in the European Union
from 2008 to Present Day. Drug Test Anal.

[10] Deventer M.
H., Stove C. P. (2025). Outsmarting
Generic Legislation: 4 Years into the Cat-and-Mouse
Game of the Synthetic Cannabinoid Receptor Agonist Market since the
Chinese Ban in 2021. Arch. Toxicol..

[11] Deventer M. H., Persson M., Norman C., Liu H., Connolly M. J., Daéid N. N., McKenzie C., Gréen H., Stove C. P. (2024). In Vitro Cannabinoid Activity Profiling of Generic
Ban-Evading Brominated Synthetic Cannabinoid Receptor Agonists and
Their Analogs. Drug Testing and Analysis.

[12] de
Godoi A. B., Zeoly L. A., Lanaro R., Oliveira I. S., Diana M. C., Yonamine M., Costa J. L. (2026). Identification and
Structural Elucidation of a New Synthetic Cannabinoid, MDMB-5′Br-PINACA,
in Seized Herbal Materials. Drug Testing and
Analysis.

[13] Daziani G., Taoussi O., Berardinelli D., Bambagiotti G., Huestis M. A., Busardò F. P., Carlier J. (2026). Comparison of Authentic
Urine N-Ethylpentedrone Metabolites to Predicted in Silico and in
Vitro Human Hepatocyte Metabolism. J. Pharm.
Biomed Anal.

[14] Godoi A. B., Antunes N. de J., Cunha K. F., Martins A. F., Huestis M. A., Costa J. L. (2024). Metabolic Stability
and Metabolite Identification of
N-Ethyl Pentedrone Using Rat, Mouse and Human Liver Microsomes. Pharmaceutics.

[15] Asha S., Vidyavathi M. (2010). Role of Human Liver Microsomes in
In Vitro Metabolism
of Drugs—A Review. Appl. Biochem. Biotechnol..

[16] Calemi D. B. de A., Godoi A. B., Minuti G., Neto F. C., Hispagnol G. F., Pilon A. C., Costa J. L., Hyslop S., Antunes N. de J. (2025). Evaluation
of Violacein Metabolic Stability and Metabolite Identification in
Human, Mouse, and Rat Liver Microsomes. Pharmaceutics.

[17] Allard S., Allard P.-M., Morel I., Gicquel T. (2019). Application of a Molecular
Networking Approach for Clinical and Forensic Toxicology Exemplified
in Three Cases Involving 3-MeO-PCP, Doxylamine, and Chlormequat. Drug Test Anal.

[18] Wang M., Carver J. J., Phelan V. V., Sanchez L. M., Garg N., Peng Y., Nguyen D. D., Watrous J., Kapono C. A., Luzzatto-Knaan T., Porto C., Bouslimani A., Melnik A. V., Meehan M. J., Liu W.-T., Crüsemann M., Boudreau P. D., Esquenazi E., Sandoval-Calderón M., Kersten R. D., Pace L. A., Quinn R. A., Duncan K. R., Hsu C.-C., Floros D. J., Gavilan R. G., Kleigrewe K., Northen T., Dutton R. J., Parrot D., Carlson E. E., Aigle B., Michelsen C. F., Jelsbak L., Sohlenkamp C., Pevzner P., Edlund A., McLean J., Piel J., Murphy B. T., Gerwick L., Liaw C.-C., Yang Y.-L., Humpf H.-U., Maansson M., Keyzers R. A., Sims A. C., Johnson A. R., Sidebottom A. M., Sedio B. E., Klitgaard A., Larson C. B., P C. A. B., Torres-Mendoza D., Gonzalez D. J., Silva D. B., Marques L. M., Demarque D. P., Pociute E., O’Neill E. C., Briand E., Helfrich E. J. N., Granatosky E. A., Glukhov E., Ryffel F., Houson H., Mohimani H., Kharbush J. J., Zeng Y., Vorholt J. A., Kurita K. L., Charusanti P., McPhail K. L., Nielsen K. F., Vuong L., Elfeki M., Traxler M. F., Engene N., Koyama N., Vining O. B., Baric R., Silva R. R., Mascuch S. J., Tomasi S., Jenkins S., Macherla V., Hoffman T., Agarwal V., Williams P. G., Dai J., Neupane R., Gurr J., Rodríguez A. M.
C., Lamsa A., Zhang C., Dorrestein K., Duggan B. M., Almaliti J., Allard P.-M., Phapale P., Nothias L.-F., Alexandrov T., Litaudon M., Wolfender J.-L., Kyle J. E., Metz T. O., Peryea T., Nguyen D.-T., VanLeer D., Shinn P., Jadhav A., Müller R., Waters K. M., Shi W., Liu X., Zhang L., Knight R., Jensen P. R., Palsson B. O., Pogliano K., Linington R. G., Gutiérrez M., Lopes N. P., Gerwick W. H., Moore B. S., Dorrestein P. C., Bandeira N. (2016). Sharing and Community Curation of Mass Spectrometry
Data with Global Natural Products Social Molecular Networking. Nat. Biotechnol..

[19] Gicquel T., Pelletier R., Richeval C., Gish A., Hakim F., Ferron P.-J., Mesli V., Allorge D., Morel I., Gaulier J.-M. (2022). Metabolite
Elucidation of 2-Fluoro-Deschloroketamine
(2F-DCK) Using Molecular Networking across Three Complementary in
Vitro and in Vivo Models. Drug Test Anal.

[20] Le
Daré B., Allard S., Bouvet R., Baert A., Allard P.-M., Morel I., Gicquel T. (2020). A Case of Fatal Acebutolol
Poisoning: An Illustration of the Potential of Molecular Networking. Int. J. Legal Med..

[21] Le
Daré B., Ferron P.-J., Couette A., Ribault C., Morel I., Gicquel T. (2021). In Vivo and in Vitro α-Amanitin
Metabolism Studies Using Molecular Networking. Toxicol. Lett..

[22] Le
Daré B., Ferron P.-J., Allard P.-M., Clément B., Morel I., Gicquel T. (2020). New Insights into Quetiapine Metabolism
Using Molecular Networking. Sci. Rep.

[23] Daina A., Michielin O., Zoete V. (2017). SwissADME: A Free Web Tool to Evaluate
Pharmacokinetics, Drug-Likeness and Medicinal Chemistry Friendliness
of Small Molecules. Sci. Rep.

[24] Daina A., Zoete V. (2016). A BOILED-Egg To Predict
Gastrointestinal Absorption and Brain Penetration
of Small Molecules. ChemMedChem..

[25] Wishart D. S., Tian S., Allen D., Oler E., Peters H., Lui V. W., Gautam V., Djoumbou-Feunang Y., Greiner R., Metz T. O. (2022). BioTransformer 3.0-a Web Server for
Accurately Predicting Metabolic Transformation Products. Nucleic Acids Res..

[26] Matlock M. K., Hughes T. B., Swamidass S. J. (2015). XenoSite
Server: A Web-Available
Site of Metabolism Prediction Tool. Bioinformatics.

[27] Jia L., Liu X. (2007). The Conduct
of Drug Metabolism Studies Considered Good Practice (II):
In Vitro Experiments. Curr. Drug Metab.

[28] Godoi A. B., Antunes N. de J., Rodrigues L. C., Martins A. F., Costa J. L. (2025). In Vitro
Metabolism and Metabolite Identification of Eutylone Using Rat Liver
Microsomes. J. Pharm. Biomed Anal.

[29] Sumner L. W., Amberg A., Barrett D., Beale M. H., Beger R., Daykin C. A., Fan T. W.-M., Fiehn O., Goodacre R., Griffin J. L., Hankemeier T., Hardy N., Harnly J., Higashi R., Kopka J., Lane A. N., Lindon J. C., Marriott P., Nicholls A. W., Reily M. D., Thaden J. J., Viant M. R. (2007). Proposed Minimum
Reporting Standards for Chemical Analysis
Chemical Analysis Working Group (CAWG) Metabolomics Standards Initiative
(MSI). Metabolomics.

[30] Tettey J. N. A., Crean C., Rodrigues J., Angeline Yap T. W., Lee Wendy Lim J., Shirley Lee H. Z., Ching M. (2021). United Nations Office
on Drugs and Crime: Recommended Methods for the Identification and
Analysis of Synthetic Cannabinoid Receptor Agonists in Seized Materials. Forensic Sci. Int. Synerg.

[31] Oashi T., Ringer A. L., Raman E. P., Mackerell A. D. (2011). Automated
Selection of Compounds with Physicochemical Properties to Maximize
Bioavailability and Druglikeness. J. Chem. Inf
Model.

[32] Lee J., Beers J. L., Geffert R. M., Jackson K. D. (2024). A Review of CYP-Mediated
Drug Interactions: Mechanisms and In Vitro Drug-Drug Interaction Assessment. Biomolecules.

[33] Brents L. K., Zimmerman S. M., Saffell A. R., Prather P. L., Fantegrossi W. E. (2013). Differential
Drug-Drug Interactions of the Synthetic Cannabinoids JWH-018 and JWH-073:
Implications for Drug Abuse Liability and Pain Therapy. J. Pharmacol Exp Ther.

[34] Martin Y. C. (2005). A Bioavailability
Score. J. Med. Chem..

[35] Lipinski C. A., Lombardo F., Dominy B. W., Feeney P. J. (2001). Experimental and
Computational Approaches to Estimate Solubility and Permeability in
Drug Discovery and Development Settings1. Adv.
Drug Delivery Rev..

[36] Meyer M. R. (2018). Toxicokinetics
of NPS: Update 2017. Handb Exp Pharmacol.

[37] Monti M. C., Rautio T., Deventer M. H., Schläpfer M., Tveit J., Krotulski A. J., Marland V., Reid R., Daeid N. N., McKenzie C., Stove C. P., Green H., Norman C. (2025). Tail-Less Precursors
in Synthetic Cannabinoid Production:
Investigating a Clandestine Laboratory, Seized Samples, and CB1 Activity. Arch. Toxicol..

[38] Kill J. B., Oliveira I. F., Tose L. V., Costa H. B., Kuster R. M., Machado L. F., Correia R. M., Rodrigues R. R. T., Vasconcellos G. A., Vaz B. G., Romão W. (2016). Chemical Characterization
of Synthetic Cannabinoids by Electrospray Ionization FT-ICR Mass Spectrometry. Forensic Sci. Int..

[39] The European Union Drugs Agency (EUDA). Synthetic Cannabinoids in Europe - a Review; 2021. https://www.euda.europa.eu/publications/rapid-communications/synthetic-cannabinoids-europe-review_en (accessed 2026-01-03).

[40] Åstrand A., Laudadio E., Gameli P. S., Martin L., Carlier J., Busardò F. P., Dahlén J., Wu X., Konradsson P., Vikingsson S., Kronstrand R., Gréen H. (2026). Structure-Activity
Relationship of Prevalent Synthetic Cannabinoid Metabolites on hCB1
in Vitro and in Silico Dynamics. Acta Pharmacol
Sin.

[41] Gamage T. F., Farquhar C. E., McKinnie R. J., Kevin R. C., McGregor I. S., Trudell M. L., Wiley J. L., Thomas B. F. (2019). Synthetic Cannabinoid
Hydroxypentyl Metabolites Retain Efficacy at Human Cannabinoid Receptors. J. Pharmacol Exp Ther.

[42] Alves V. L., Gonçalves J. L., Aguiar J., Teixeira H. M., Câmara J. S. (2020). The Synthetic
Cannabinoids Phenomenon: From Structure to Toxicological Properties.
A Review. Crit Rev. Toxicol.

[43] Xiang J., Wen D., Zhao J., Xiang P., Shi Y., Ma C. (2023). Study of the
Metabolic Profiles of “Indazole-3-Carboxamide” and “Isatin
Acyl Hydrazone” (OXIZID) Synthetic Cannabinoids in a Human
Liver Microsome Model Using UHPLC-QE Orbitrap MS. Metabolites.

[44] Rautio T., Obrist R., Krebs L., Klingstedt T., Dahlén J., Wu X., Gréen H. (2025). In Vitro Metabolism
Study of ADB-P-5Br-INACA and ADB-4en-P-5Br-INACA Using Human Hepatocytes,
Liver Microsomes, and in-House Synthesized References. Drug Test Anal.

[45] Erol
Ozturk Y., Yeter O. (2021). In Vitro Phase I Metabolism of the
Recently Emerged Synthetic MDMB-4en-PINACA and Its Detection in Human
Urine Samples. J. Anal Toxicol.

[46] Riedmaier S., Klein K., Winter S., Hofmann U., Schwab M., Zanger U. M. (2011). Paraoxonase (PON1
and PON3) Polymorphisms: Impact on
Liver Expression and Atorvastatin-Lactone Hydrolysis. Front Pharmacol.

[47] Carlier J., Diao X., Sempio C., Huestis M. A. (2017). Identification of
New Synthetic Cannabinoid ADB-CHMINACA (MAB-CHMINACA) Metabolites
in Human Hepatocytes. AAPS J..

[48] Fabregat-Safont D., Mata-Pesquera M., Barneo-Muñoz M., Martinez-Garcia F., Mardal M., Davidsen A. B., Sancho J. V., Hernández F., Ibáñez M. (2022). In-Depth Comparison
of the Metabolic and Pharmacokinetic
Behaviour of the Structurally Related Synthetic Cannabinoids AMB-FUBINACA
and AMB-CHMICA in Rats. Commun. Biol..

[49] Brents L. K., Reichard E. E., Zimmerman S. M., Moran J. H., Fantegrossi W. E., Prather P. L. (2011). Phase I Hydroxylated
Metabolites of the K2 Synthetic
Cannabinoid JWH-018 Retain In Vitro and In Vivo Cannabinoid 1 Receptor
Affinity and Activity. PLoS One.

[50] Ahmed
Laskar A., Younus H. (2019). Aldehyde Toxicity and Metabolism:
The Role of Aldehyde Dehydrogenases in Detoxification, Drug Resistance
and Carcinogenesis. Drug Metab Rev..

