# Supplementary material for: Physicochemical Characterization and Metabolites Identification of the Synthetic Cannabinoid MDMB-5′Br-PINACA Using In Silico and In Vitro Approaches
Source: Chem Res Toxicol. 2026 May 24;39(6):1190–206. doi: 10.1021/acs.chemrestox.6c00107 (PMC13273808; doi:10.1021/acs.chemrestox.6c00107)
Supplement: Supplementary file 1 [file tx6c00107_si_001.pdf]

Supporting Information

Physicochemical characterization and metabolites identification of the synthetic cannabinoid MDMB-5′Br-PINACA using in silico and in vitro approaches

Alexandre B. Godoi[†,‡], Natalícia J. Antunes[*§], Júlio C. C. da Silva[‖], Gabriel Cordeiro[†,‡], Tássia F. D. Castro[†,‡], José L. Costa[†, §]
[†] Campinas Poison Control Center, Universidade Estadual de Campinas (UNICAMP), Campinas, SP 13083-859, Brazil
[‡] School of Medical Sciences, Universidade Estadual de Campinas (UNICAMP), Campinas, SP 13083-859, Brazil
[§] Faculty of Pharmaceutical Sciences, Universidade Estadual de Campinas (UNICAMP), Campinas, SP 13083-859, Brazil
[‖] Nova Analítica Imp. Exp. LTDA, Sao Paulo, SP 09941-202, Brazil

[*]**Corresponding author**: Faculty of Pharmaceutical Sciences, Universidade Estadual de Campinas (UNICAMP), R. Cândido Portinari, 200, Cidade Universitária, 13083-871, Campinas, SP, Brazil.
E-mail address: natja@unicamp.br (Antunes, N.J.)
Tel.: +55-19-3521-8181
ORCID: https://orcid.org/0000-0002-7820-552X

FIGURES

Figure S1. MS^1-mass spectra of MDMB-5'Br-PINACA metabolites considering phase I metabolism in pooled human liver microsomes (pHLM) by LC-HRMS using electrospray ionization in positive mode (ESI^+)……………………………………………………………..….3

Figure S2. MS^2-mass spectra of MDMB-5'Br-PINACA metabolites considering phase I metabolism in pooled human liver microsomes (pHLM) by LC-HRMS using electrospray ionization in positive mode (ESI^+)……………………………………………………………10

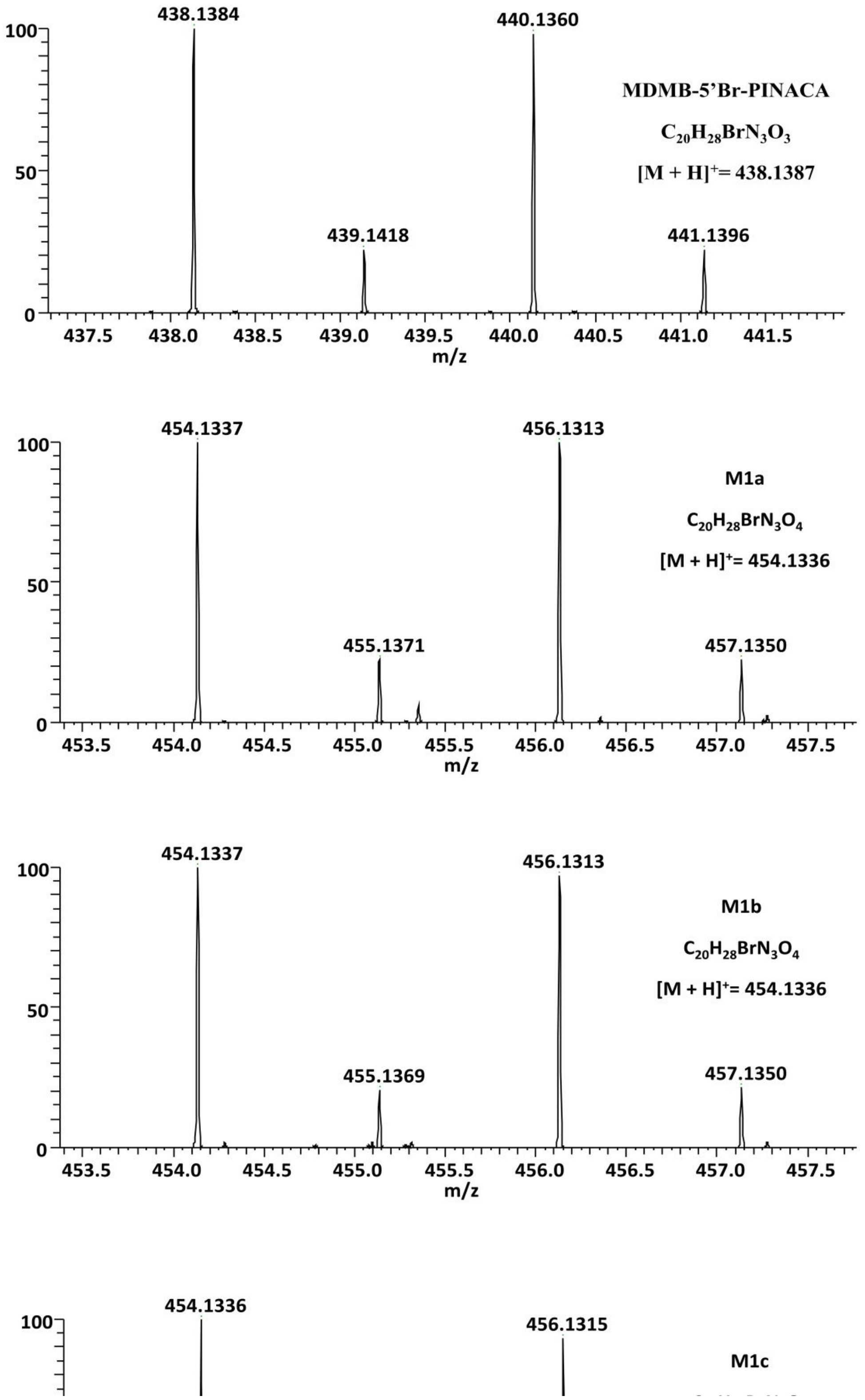

Figure S1. MS^1-mass spectra of MDMB-5'Br-PINACA metabolites considering phase I metabolism in pooled human liver microsomes (pHLM) by LC-HRMS using electrospray ionization in positive mode (ESI^+).

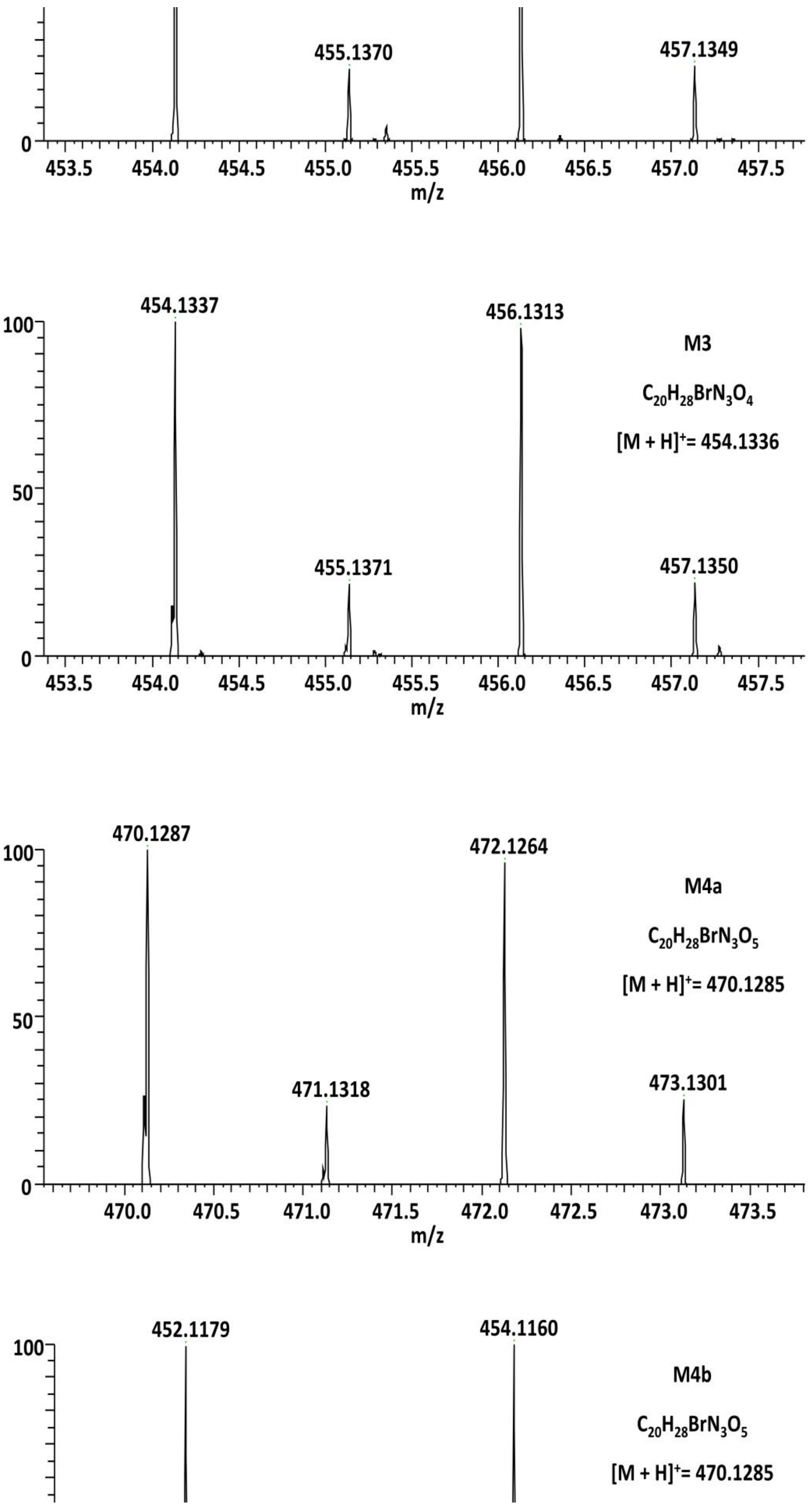

Figure S1 (Continuation). MS[1]-mass spectra of MDMB-5’Br-PINACA metabolites considering phase I metabolism in pooled human liver microsomes (pHLM) by LC-HRMS using electrospray ionization in positive mode (ESI^+).

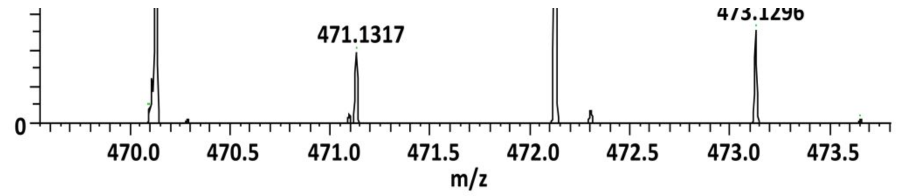

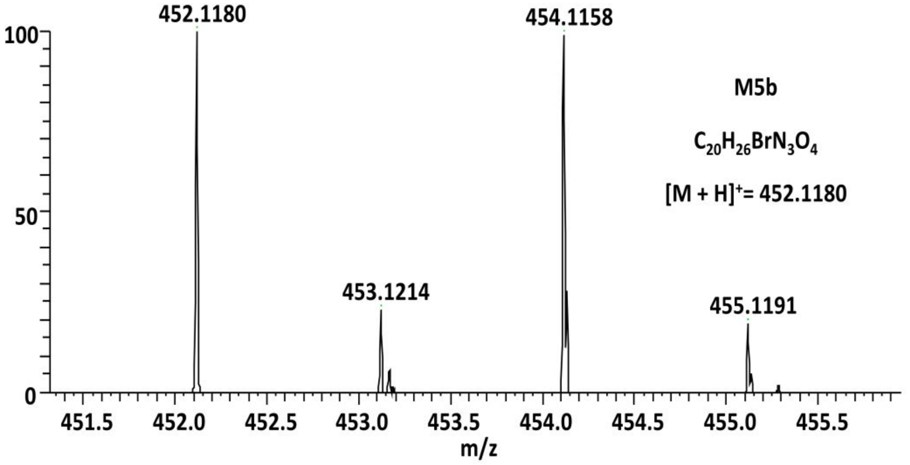

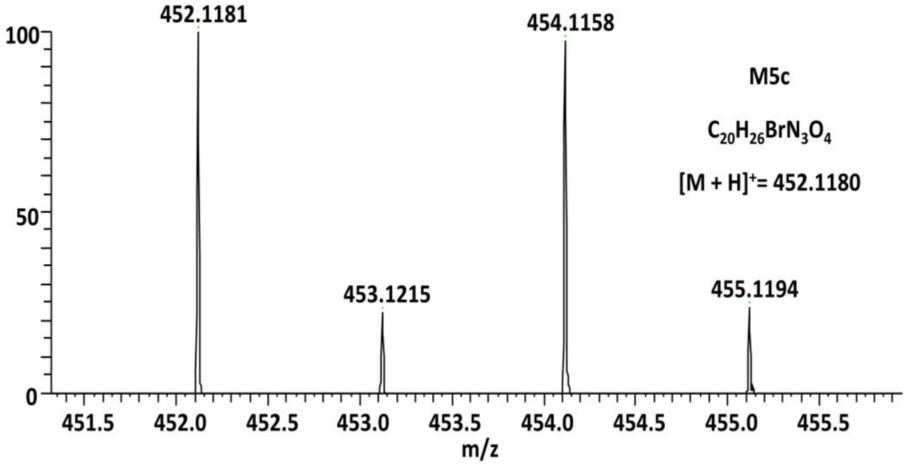

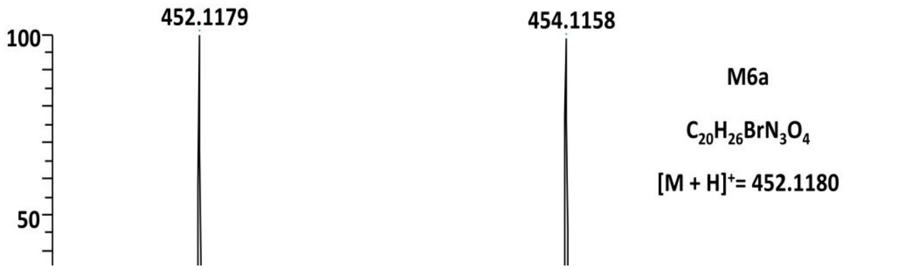

Figure S1 (Continuation). MS^1-mass spectra of MDMB-5'Br-PINACA metabolites considering phase I metabolism in pooled human liver microsomes (pHLM) by LC-HRMS using electrospray ionization in positive mode (ESI^+).

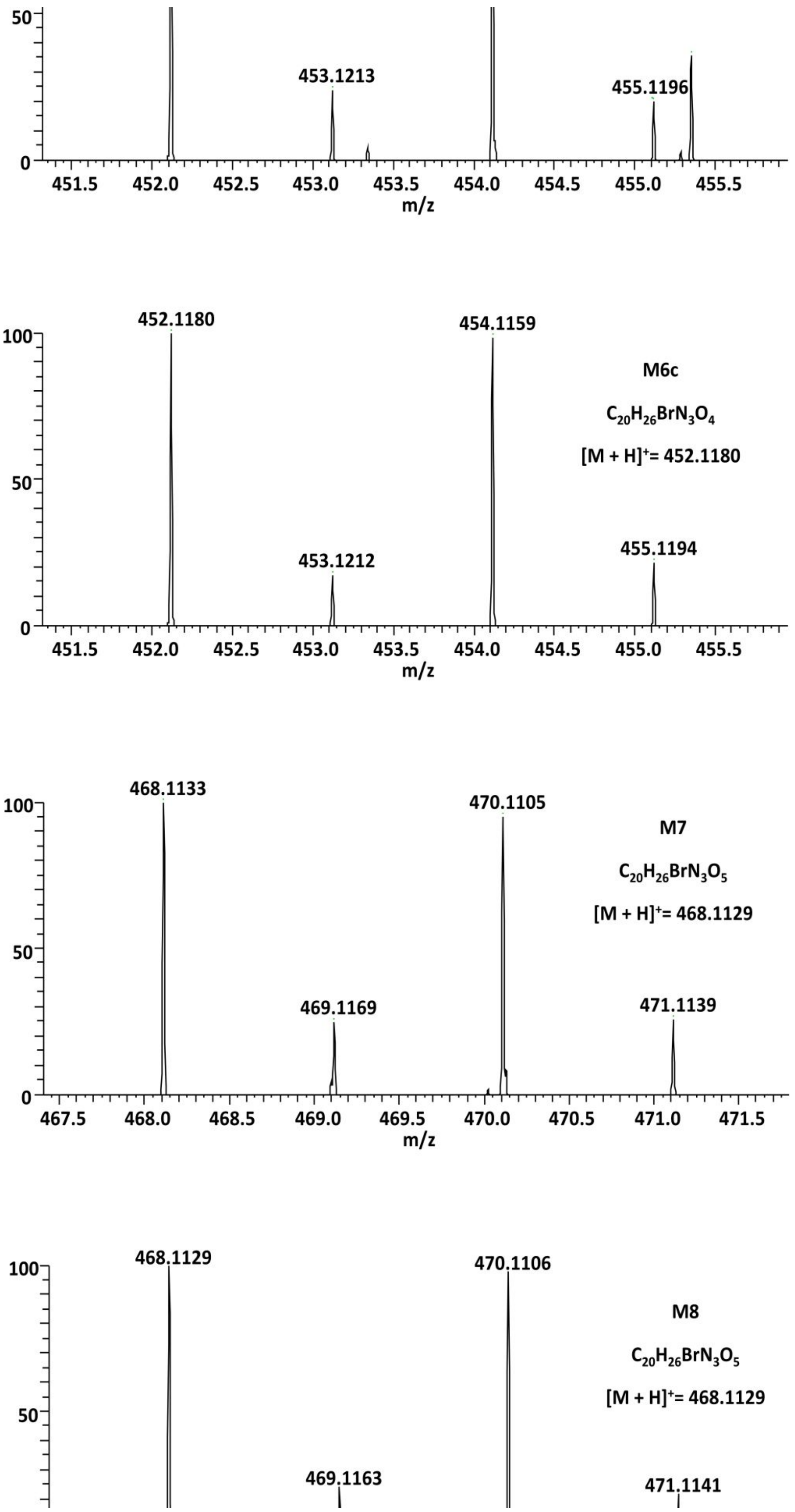

Figure S1 (Continuation). MS^1-mass spectra of MDMB-5'Br-PINACA metabolites considering phase I metabolism in pooled human liver microsomes (pHLM) by LC-HRMS using electrospray ionization in positive mode (ESI^+).

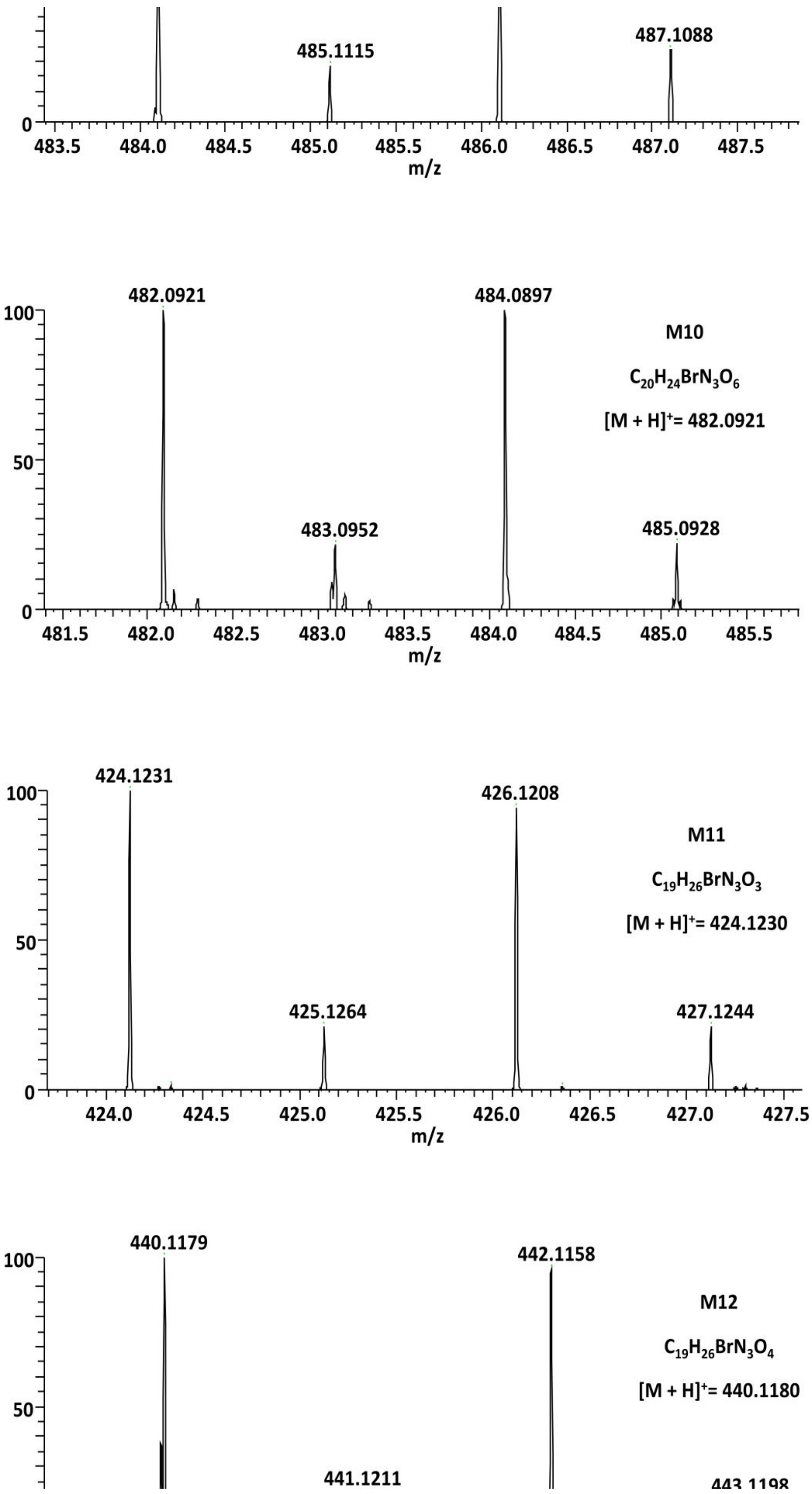

Figure S1 (Continuation). MS[1]-mass spectra of MDMB-5'Br-PINACA metabolites considering phase I metabolism in pooled human liver microsomes (pHLM) by LC-HRMS using electrospray ionization in positive mode (ESI+).

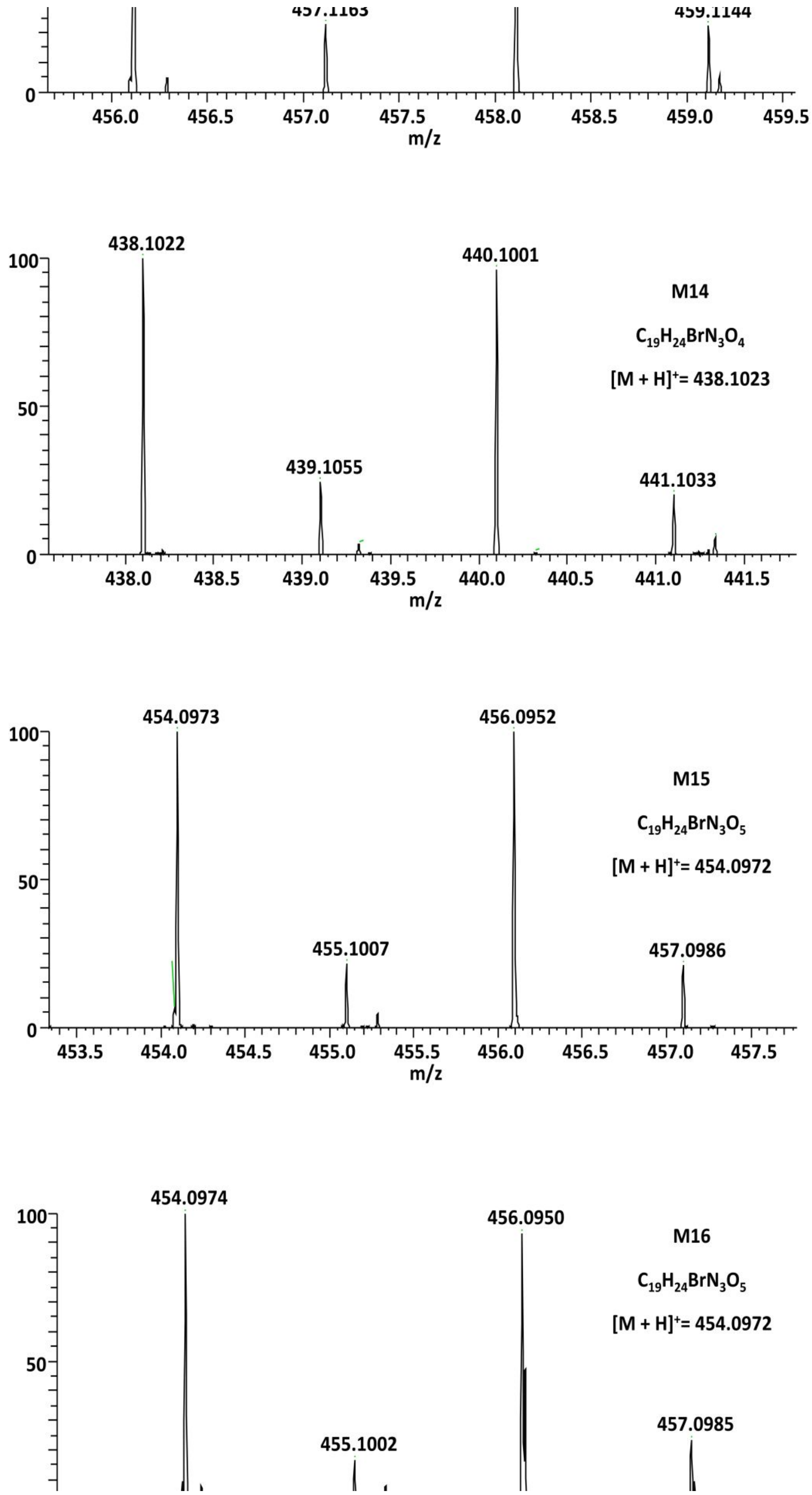

Figure S1 (Continuation). MS[1]-mass spectra of MDMB-5'Br-PINACA metabolites considering phase I metabolism in pooled human liver microsomes (pHLM) by LC-HRMS using electrospray ionization in positive mode (ESI$^+$).

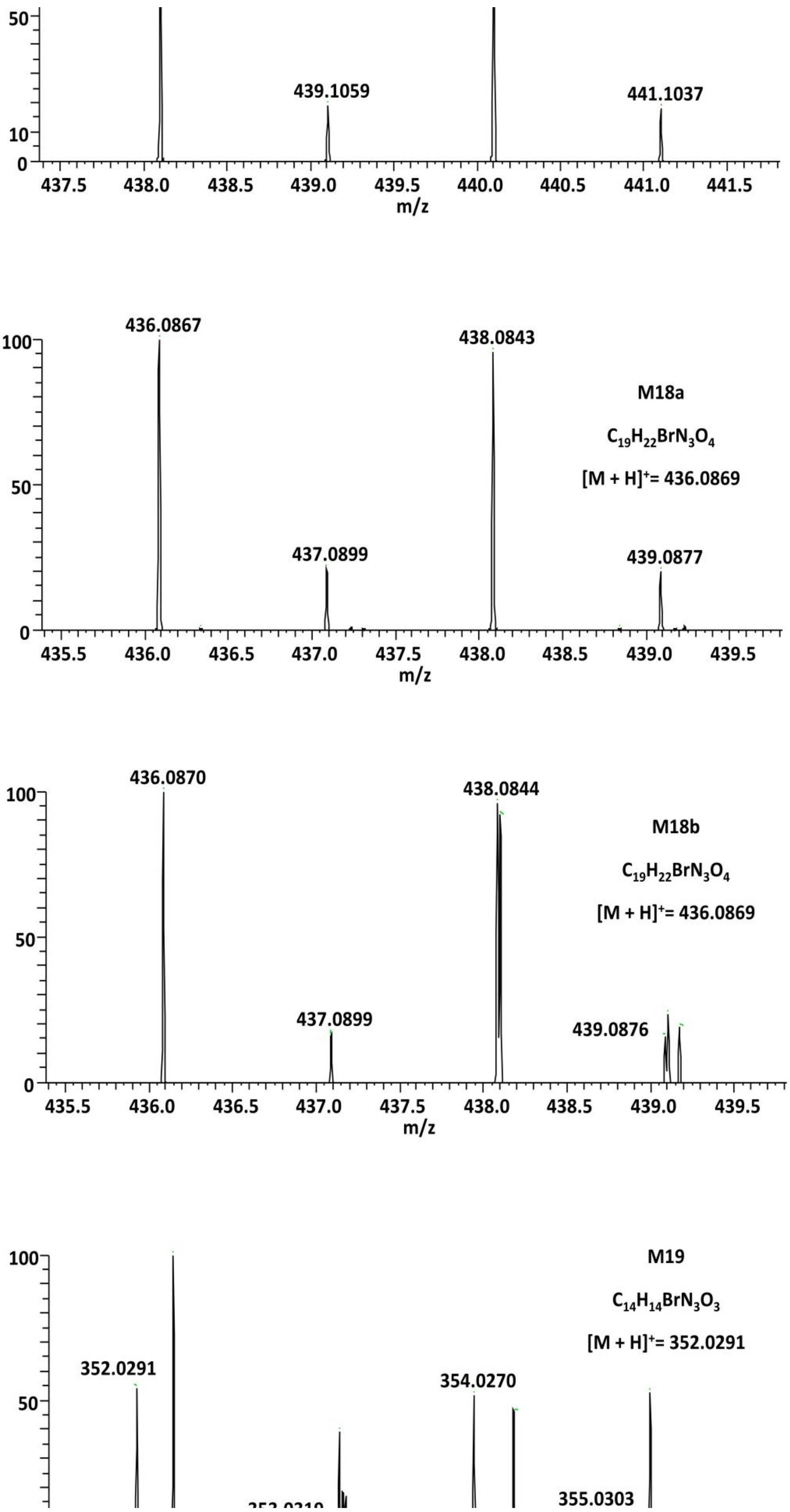

Figure S1 (Continuation). MS[1]-mass spectra of MDMB-5'Br-PINACA metabolites considering phase I metabolism in pooled human liver microsomes (pHLM) by LC-HRMS using electrospray ionization in positive mode (ESI+).

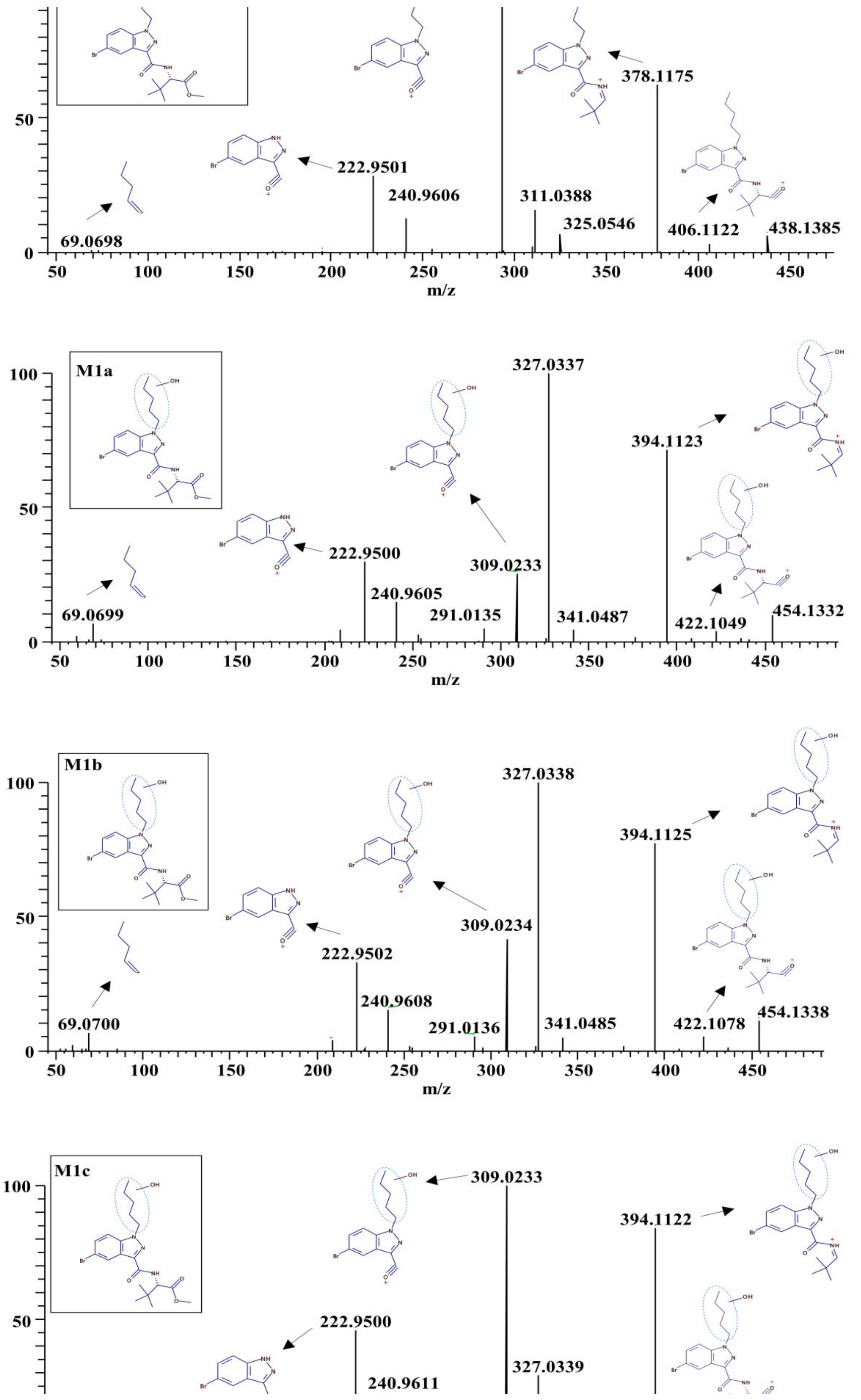

Figure S2. MS^2-mass spectra of MDMB-5'Br-PINACA metabolites considering phase I metabolism in pooled human liver microsomes (pHLM) by LC-HRMS using electrospray ionization in positive mode (ESI^+).

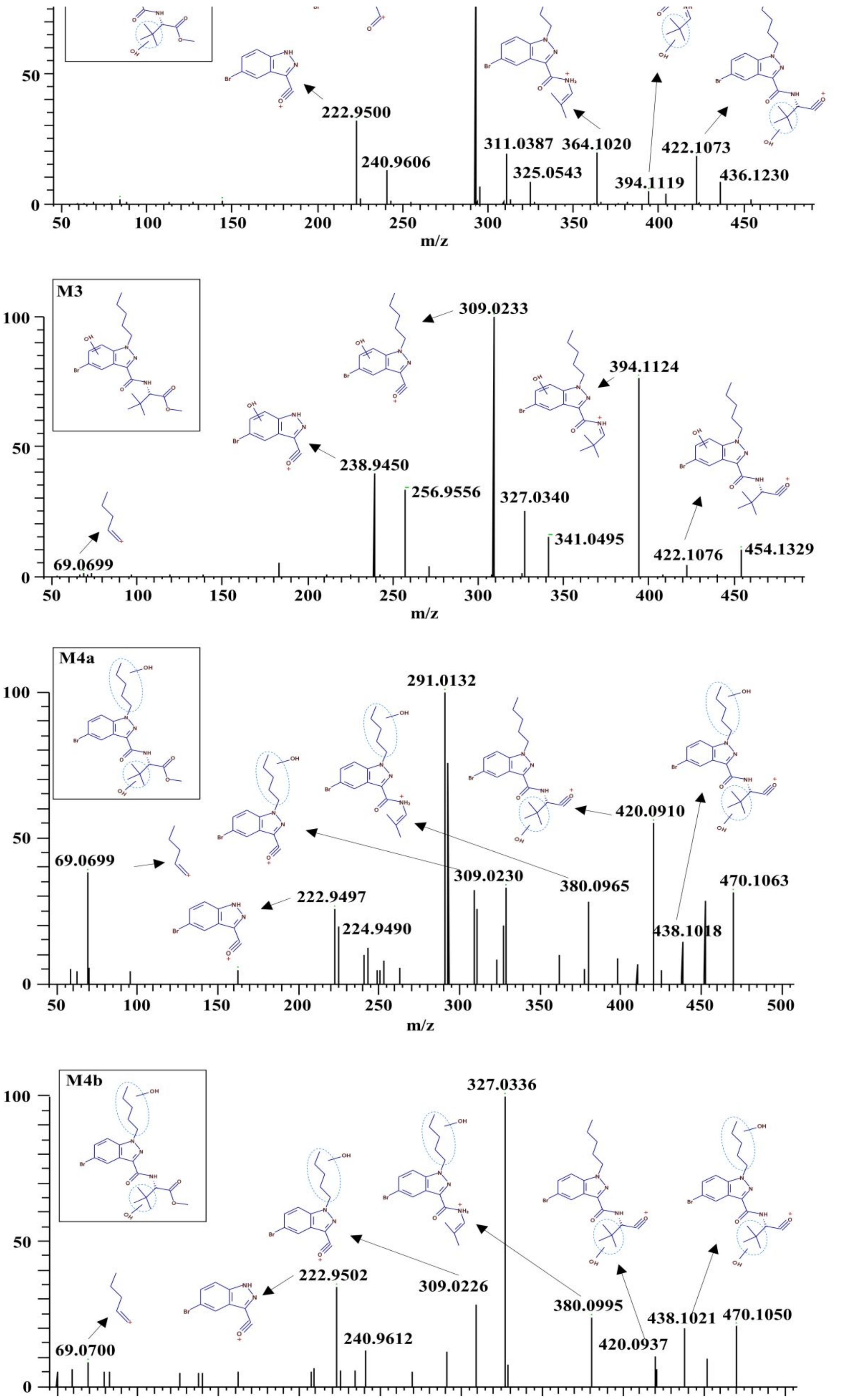

Figure S2 (Continuation). MS^2-mass spectra of MDMB-5'Br-PINACA metabolites considering phase I metabolism in pooled human liver microsomes (pHLM) by LC-HRMS using electrospray ionization in positive mode (ESI^+).

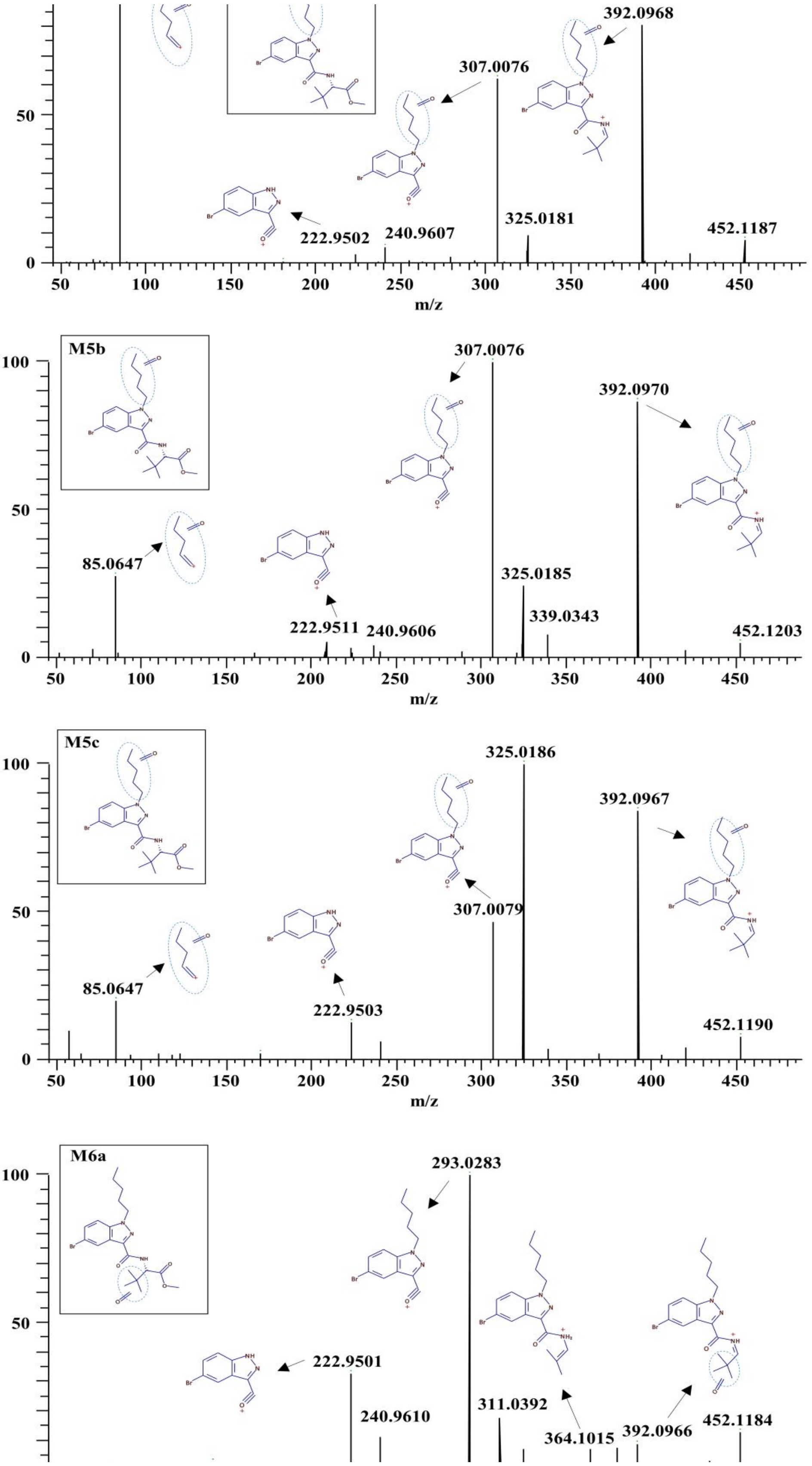

Figure S2 (Continuation). MS^2-mass spectra of MDMB-5'Br-PINACA metabolites considering phase I metabolism in pooled human liver microsomes (pHLM) by LC-HRMS using electrospray ionization in positive mode (ESI^+).

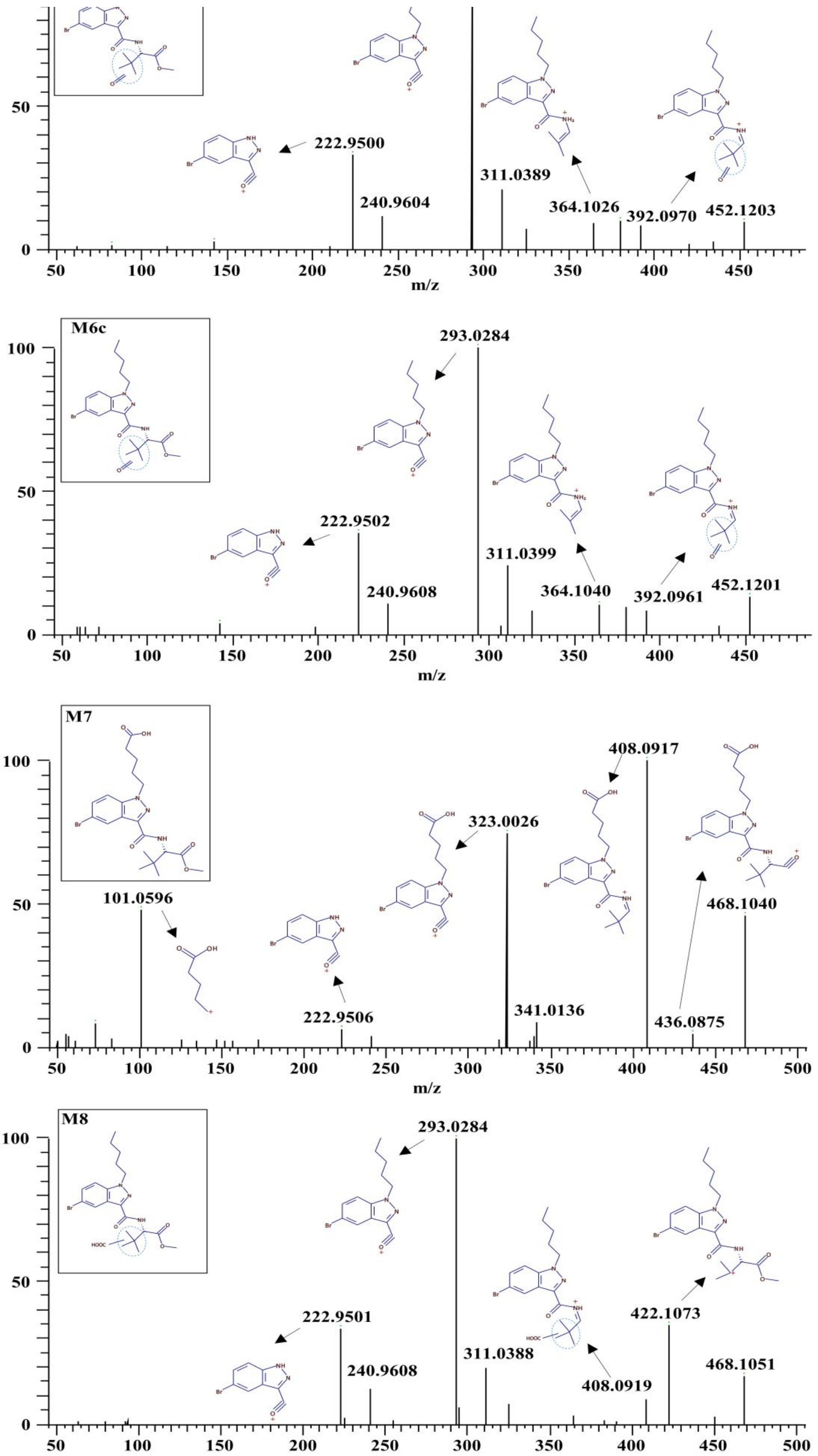

Figure S2 (Continuation). MS^2-mass spectra of MDMB-5'Br-PINACA metabolites considering phase I metabolism in pooled human liver microsomes (pHLM) by LC-HRMS using electrospray ionization in positive mode (ESI$^+$).

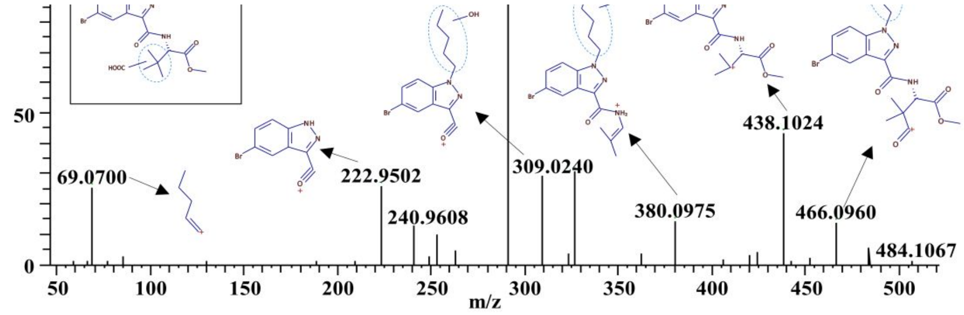

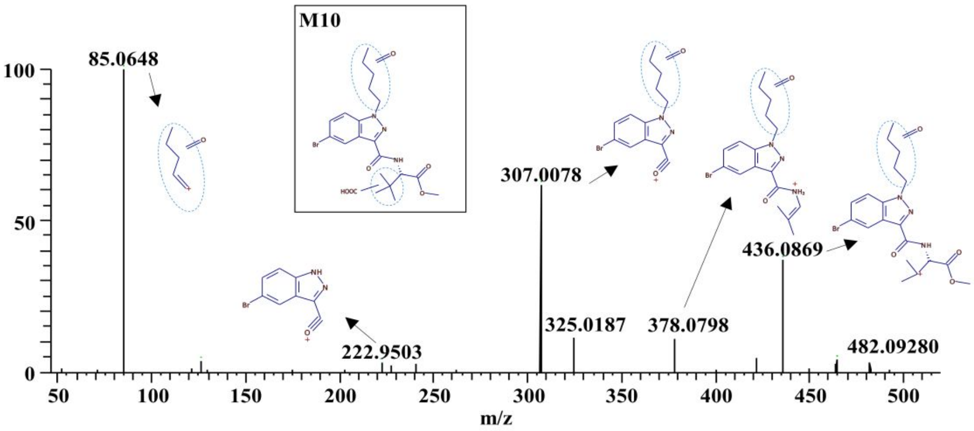

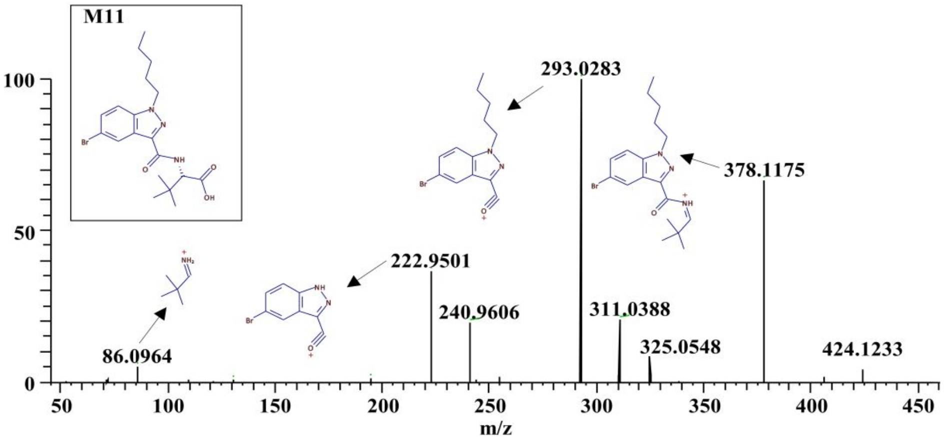

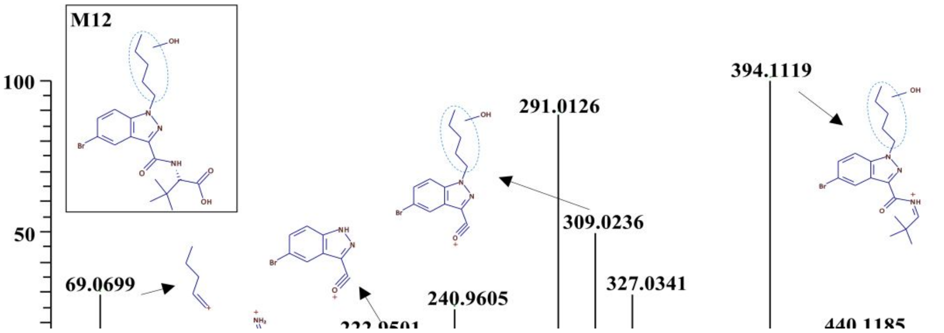

Figure S2 (Continuation). MS^2-mass spectra of MDMB-5'Br-PINACA metabolites considering phase I metabolism in pooled human liver microsomes (pHLM) by LC-HRMS using electrospray ionization in positive mode (ESI^+).

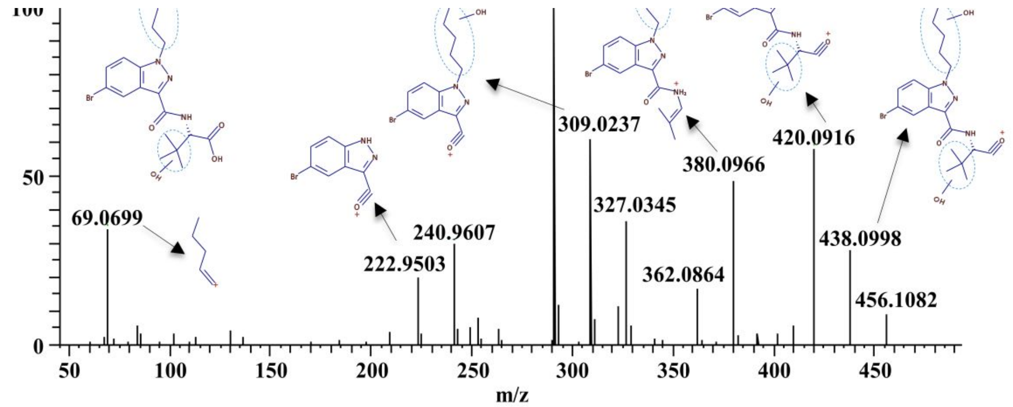

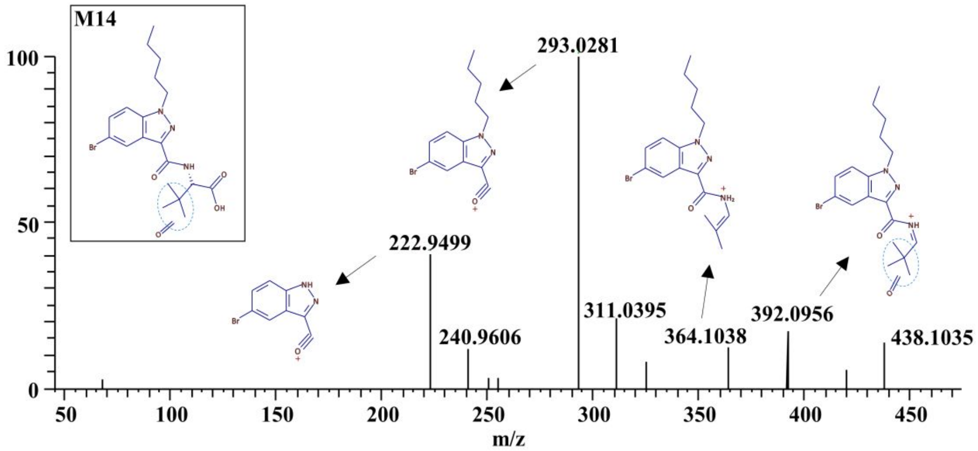

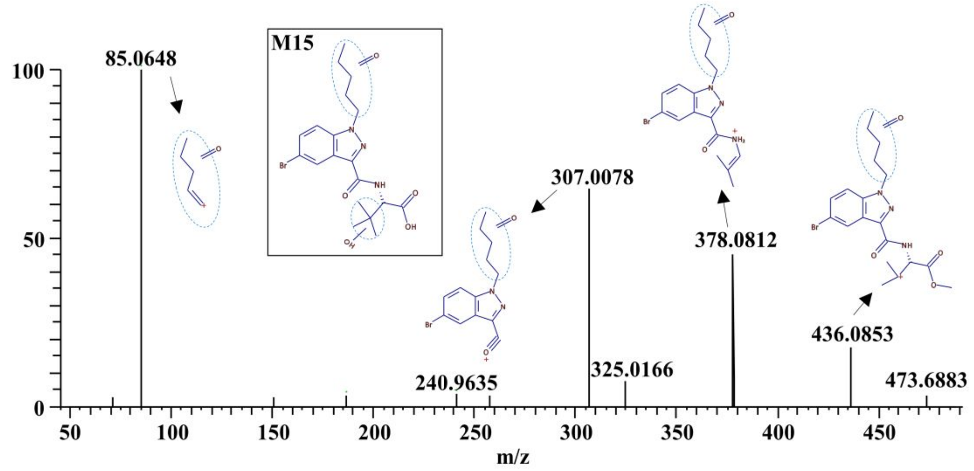

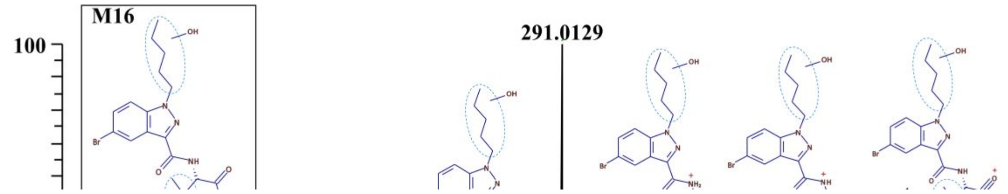

Figure S2 (Continuation). MS^2-mass spectra of MDMB-5'Br-PINACA metabolites considering phase I metabolism in pooled human liver microsomes (pHLM) by LC-HRMS using electrospray ionization in positive mode (ESI^+).

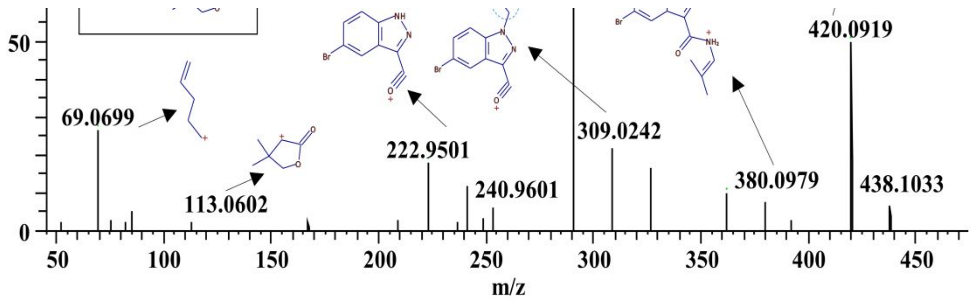

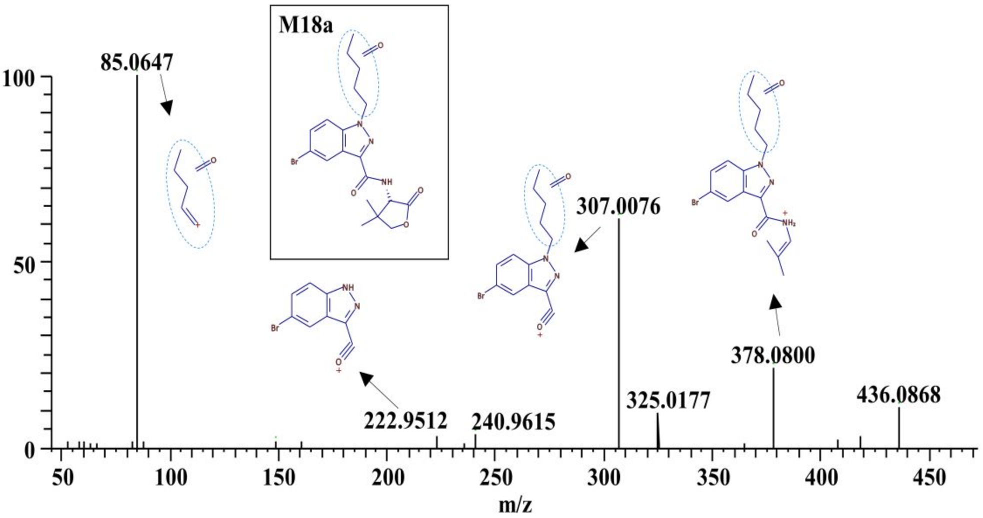

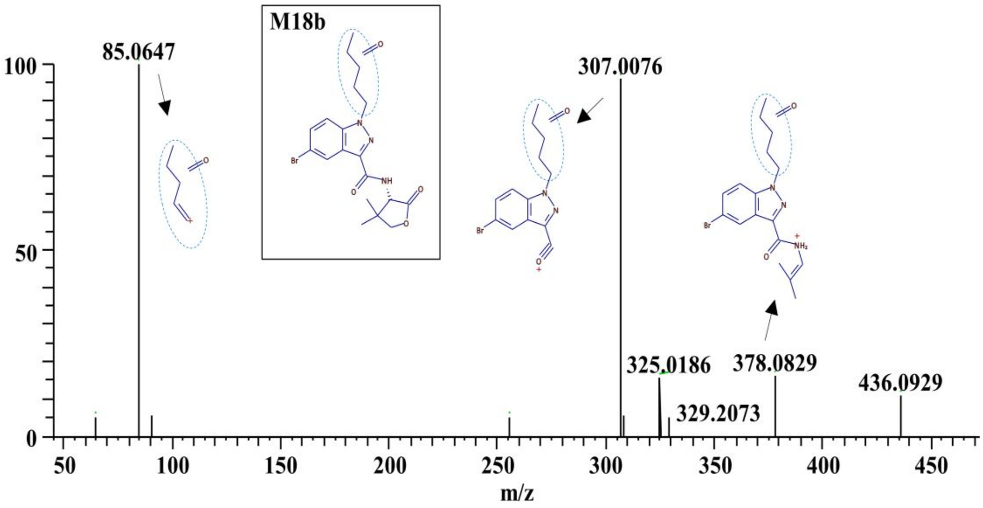

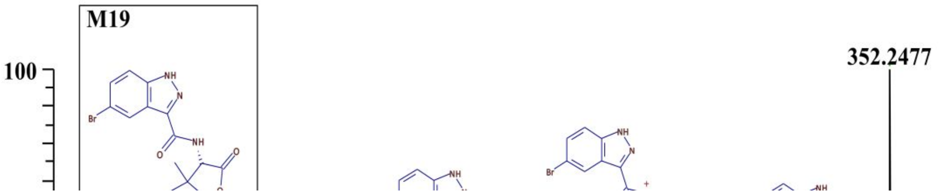

Figure S2 (Continuation). MS^2-mass spectra of MDMB-5'Br-PINACA metabolites considering phase I metabolism in pooled human liver microsomes (pHLM) by LC-HRMS using electrospray ionization in positive mode (ESI$^+$).
